# Vertical Graphene Nanowalls Anchored on Ti_3_C_2_T_*x*_ MXene as a Hierarchical Composite with Enhanced Supercapacitive Performance

**DOI:** 10.1021/acsami.6c04344

**Published:** 2026-06-26

**Authors:** Yang Ma, Ghulam Farid, Shubhadeep Majumdar, Enric Bertran-Serra, Jarosław Serafin, Stefanos Chaitoglou, Roger Amade-Rovira

**Affiliations:** † Department of Applied Physics, 16724 University of Barcelona, C/Martí i Franquès, 1, 08028 Barcelona, Catalunya, Spain; ‡ ENPHOCAMAT Group, Institute of Nanoscience and Nanotechnology (IN2UB), University of Barcelona, C/Martí i Franquès, 1, 08028 Barcelona, Catalunya, Spain; § Department of Inorganic and Organic Chemistry, Inorganic Chemistry Section, University of Barcelona, Martí i Franquès 1-11, 08028 Barcelona, Spain

**Keywords:** Ti_3_C_2_T_*x*_ MXene, vertical graphene, hierarchical nanostructure, ICP-CVD, supercapacitive performance

## Abstract

MXene composites have gained considerable attention for their potential application in energy storage devices. An important limitation arises from the fact that MXene nanosheets are prone to restacking and self-aggregation during electrochemical operation, which reduces electrolyte accessibility and limits practical charge storage. This limitation can be mitigated by introducing suitable intercalation scaffolds. In this work, a hierarchical architecture is developed by integrating vertical graphene nanowalls (GNWs) with Ti_3_C_2_T_*x*_ MXene. The GNWs/Ti_3_C_2_T_*x*_ hybrid structure is fabricated through a stepwise route involving selective acid etching of Ti_3_AlC_2_, film assembly by filtration, and subsequent growth of GNWs via inductively coupled plasma chemical vapor deposition (ICP-CVD). Owing to their large accessible surface area and rigid vertical morphology, GNWs act as effective spacers that stabilize the Ti_3_C_2_T_*x*_ interlayer framework, expand the intersheet spacing, and create multidirectional, continuous ion-transport pathways. Consequently, a greater fraction of ion-accessible electroactive sites is exposed and utilized more efficiently. The electrochemical performance of the GNWs/Ti_3_C_2_T_*x*_ electrode was evaluated in aqueous H_2_SO_4_ within a 0.7 V potential window. At a scan rate of 10 mV s^–1^, the hybrid delivers an areal capacitance of 163.17 mF cm^–2^, corresponding to a 1.2-fold enhancement relative to Ti_3_C_2_T_*x*_ and a 7.3-fold increase compared with GNWs on graphite sheets. These results demonstrate that the GNWs/Ti_3_C_2_T_*x*_ combines structural stability with improved charge-storage kinetics, highlighting its promise for high-performance supercapacitors. More broadly, the design strategy presented here offers a practical framework for enhancing the electrochemical properties of MXene-based composites and can be extended to other hybrid electrode systems.

## Graphical abstract

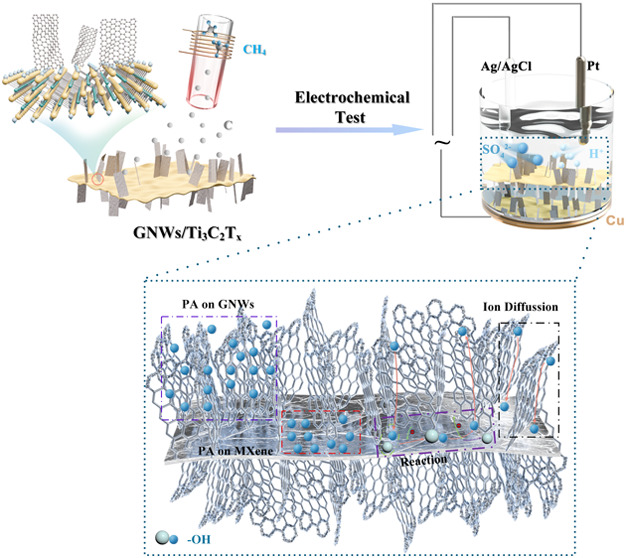

## 1 Introduction

The accelerating adoption of electric vehicles, the proliferation of portable electronic devices, and the growing prevalence of distributed sensing applications are collectively driving a significant rise in demand for electrochemical energy storage systems that can simultaneously deliver high power and extended operational lifetimes.^1,2^ Supercapacitors are of interest in this context due to their ability to facilitate rapid charging and discharging, as well as their notable cycling durability.^3–5^ These characteristics make them well-suited for integration into hybrid power architectures, complementing battery functionality. However, their practical deployment is constrained by the inherent trade-off between energy and power densities, which is largely governed by electrode composition, accessible surface area, and the efficiency of coupled ion/electron transport within the porous architecture.^6–8^

The phenomenon of charge storage in supercapacitors is predominantly attributed to two distinct mechanisms: electric double-layer capacitance (EDLC) and pseudocapacitance. Carbon-based materials like activated carbon, graphene, and carbon nanotubes have been demonstrated to facilitate EDLC through electrostatic charge accumulation at the electrode/electrolyte interface.^9–11^ They are highly valued for their chemical stability and excellent electrical conductivity.^12^ Nevertheless, purely EDLC-based electrodes often exhibit limited capacitance because the charge is stored non-Faradaically and is restricted by the accessible interfacial area and the electrolyte’s ability to penetrate the porous network.^13,14^ In contrast, pseudocapacitive materials have been shown to store charge via fast surface or near-surface Faradaic reactions, with the potential to deliver higher capacitance.^15^ However, these materials may also exhibit lower conductivity, kinetic limitations, or structural degradation during long-term cycling.^16,17^ Recent studies further demonstrate that rational electrode design often relies on balancing these two charge-storage mechanisms. Porous biomass- or biochar-derived carbons are widely investigated as EDLC-type electrodes because their hierarchical pore structures and large accessible surfaces favor electrostatic ion adsorption. In contrast, transition-metal compounds and hybrid architectures can introduce Faradaic redox reactions, thereby increasing pseudocapacitive contribution and improving energy output. These developments indicate that combining conductive carbon frameworks with redox-active components is an effective route to improve both charge-storage capacity and rate performance.^18–20^ Engineering hybrid electrodes that integrate conductive carbon frameworks with redox-active components is therefore a widely adopted strategy for reconciling high capacitance with rapid kinetics and mechanical robustness.^21^ Among emerging pseudocapacitive materials, 2D transition-metal carbides and nitrides (MXenes) have attracted substantial attention as supercapacitor electrodes due to their metallic conductivity, hydrophilicity, and rich surface chemistry.^22,23^ Ti_3_C_2_T_*x*_, one of the most widely investigated MXenes, is commonly produced by selectively etching the Al layers from Ti_3_AlC_2_ followed by delamination, yielding highly conductive nanosheets terminated with surface functional groups (T_*x*_, commonly –O/–OH/–F).^24^ Beyond supercapacitors, MXenes have also shown broad potential in diverse energy-storage and energy-conversion systems because of their metallic conductivity, hydrophilic surface chemistry, tunable interlayer spacing, and abundant surface-active sites. Recent studies have demonstrated the usefulness of MXene-based architectures in Li–S batteries and emerging energy-harvesting systems, further highlighting the versatility of MXene chemistry and structure in energy-related applications.^25,26^ These advances indicate that rational regulation of MXene surface chemistry and interlayer architecture is essential for improving electrochemical performance. These terminations promote strong electrolyte affinity and enable fast proton-coupled electron transfer in acidic media, thereby contributing to pronounced pseudocapacitive behavior.^27,28^ Despite these merits, Ti_3_C_2_T_*x*_ electrodes frequently exhibit rapid performance decay or suboptimal rate capability in practical configurations. A central limitation is the tendency of Ti_3_C_2_T_*x*_ nanosheets to restack and self-aggregate during processing and electrochemical operation, which narrows interlayer space, increases ion-transport tortuosity, and reduces the fraction of electrochemically accessible active sites. Concurrently, repeated ion insertion/extraction and interfacial polarization can exacerbate contact-resistance growth and structural densification, further limiting charge utilization at high rates. To address these challenges, the introduction of conductive intercalation scaffolds has been proposed as an effective approach to stabilize Ti_3_C_2_T_*x*_ interlayer spacing and to construct open ion-transport pathways.^29,30^ Graphene-based materials combine high electrical conductivity, chemical stability, and large specific surface area, making them well-suited as spacer frameworks to mitigate Ti_3_C_2_T_*x*_ restacking and interlayer aggregation.^31,32^ However, most reported MXene/graphene composites rely on solution-phase assembly, which may lead to microstructural inhomogeneity, limited out-of-plane conductivity, and weak interfacial coupling between components. Additionally, graphene sheets are prone to restacking, progressively diminishing access to internal surfaces during prolonged operation.^33^ These constraints motivate alternative architectural designs that simultaneously sustain continuous electronic percolation and preserve a high density of open transport channels. Vertically aligned MXene-based electrodes have been reported as an effective route to reduce restacking-induced transport limitations. For example, vertically aligned Ti_3_C_2_T_*x*_/V_2_O_5_ heterostructures and vertically aligned Ti_3_C_2_T_*x*_ electrodes have been shown to improve ion accessibility and shorten diffusion pathways compared with densely stacked MXene films.^34,35^ These studies demonstrate that out-of-plane structural design is an important strategy for improving the electrochemical utilization of MXene sheets. Subsequent studies have shown that reorienting graphene from a predominantly horizontal configuration to a vertically aligned architecture can markedly improve capacitive performance by increasing electrolyte-accessible surface area and reducing ion-transport tortuosity.^36–39^ When integrated with Ti_3_C_2_T_*x*_, this out-of-plane morphology further mitigates MXene restacking, while the interconnected carbon network facilitates efficient electron transport across the electrode thickness. GNWs can be directly grown by inductively coupled plasma chemical vapor deposition, in which methane-derived reactive carbon species are generated in the plasma phase and deposited on a heated substrate, while plasma-assisted nucleation and edge-oriented growth favor the formation of vertically aligned graphene sheets rather than compact planar carbon films.^40–43^ This catalyst-free route enables conformal integration of GNWs with Ti_3_C_2_T_*x*_/Papyex, providing a conductive out-of-plane scaffold that suppresses MXene restacking and preserves electrolyte-accessible channels. Consequently, GNWs function not only as highly conductive scaffolds but also as effective spacers that maintain electrolyte-accessible channels and establish multidirectional ion-transport pathways throughout the hybrid electrode.^44,45^

In this work, we develop a hierarchical GNWs/Ti_3_C_2_T_*x*_ architecture by anchoring GNWs onto Ti_3_C_2_T_*x*_ films assembled on a Papyex carbon substrate via the following process comprising selective etching/delamination, film fabrication, and subsequent GNWs growth by ICP-CVD. This electrode design pursues three interrelated aims: mitigating MXene restacking while preserving open interlayer spacing for efficient ion access; constructing a three-dimensional conductive framework that electrically bridges adjacent Ti_3_C_2_T_*x*_ domains; and improving wettability and electrolyte penetration through a capillary-active porous network. The study further highlights structure–property relationships in the GNWs/Ti_3_C_2_T_*x*_ system by correlating comprehensive structural and surface characterizations with electrochemical analyses. This integrated approach elucidates the respective contributions of EDLC and termination-driven pseudocapacitance in H_2_SO_4_ and provides broader design principles for engineering high-performance 2*D*/3D hybrid electrodes.

## 2 Materials and Methods

### 2.1 Preparation of MXene Nanosheets

Ti_3_C_2_T_*x*_ MXene was synthesized from Ti_3_AlC_2_ (NanoGraphi, Turkey) by selectively removing the Al layers via HF etching. Briefly, 10 mL of 48 wt % HF was placed in an HF-resistant polypropylene container, and 1 g of Ti_3_AlC_2_ powder was gradually introduced under continuous stirring to moderate the exothermic reaction and ensure uniform etching. The mixture was maintained at 30 °C for 24 h to complete Al removal. After etching, the resulting suspension was centrifuged at 3500–4000 rpm for 5–10 min (FUGELAB-GB10) to collect the Ti_3_C_2_T_*x*_ solid, followed by repeated washing with deionized water and centrifugation until the supernatant reached near-neutral pH (6–7). To obtain delaminated single- or few-layer Ti_3_C_2_T_*x*_ flakes, the washed MXene was sonicated in deionized water for 1 h using a SONICS Vibra-Cell VCX 500, promoting exfoliation into a stable colloidal suspension. The suspension was subsequently centrifuged to remove residual large aggregates, yielding a supernatant enriched in uniformly dispersed Ti_3_C_2_T_*x*_ flakes. Additional experimental details are provided in our previous work.^46^

### 2.2 Synthesis of GNWs on Ti_3_C_2_T_*x*_/Papyex

GNWs/Ti_3_C_2_T_*x*_ hybrid electrodes were fabricated by combining slurry casting with subsequent ICP-CVD growth. Ti_3_C_2_T_*x*_ films were first prepared by dispersing Ti_3_C_2_T_*x*_ powder, acetylene black, and poly­(vinylidene fluoride) (PVDF) at a mass ratio of 80:10:10 in *N*-methyl-2-pyrrolidone (NMP) to form a homogeneous slurry. The slurry was coated onto Papyex sheets (∼35 × 40 mm^2^), followed by drying at 80 °C for 12 h. The final Ti_3_C_2_T_*x*_-based coating mass loading was approximately 7.1 mg cm^–2^. GNWs were subsequently grown directly on the Ti_3_C_2_T_*x*_-coated Papyex by ICP-CVD (as shown in Figure 1). The coated substrates were mounted on a graphite holder in a reactor system consisting of a quartz tube, a 13.56 MHz RF resonator (440 W) for remote plasma generation, and a tubular furnace. The samples were placed ∼30 cm downstream from the plasma region and heated to 750 °C. After evacuation to a base pressure of 3.0 × 10^–3^ Pa using a turbomolecular pump, GNWs growth was initiated by introducing CH_4_ (10 sccm) under plasma conditions of 400 W RF power and 400 mTorr, and maintained for 30 min. Upon completion, the reactor was cooled to room temperature under vacuum. To improve surface wettability, the as-grown GNWs were finally subjected to a brief O_2_ plasma treatment at 40 W and 400 mTorr for 30 s. The ICP-CVD equipment has been shown in Figure S1, and more details on the GNWs growth can be found in our previous reports.^41–43^ In this research, Ti_3_AlC_2_ and Ti_3_C_2_T_*x*_ will first be coated onto the Papyex substrate, labeled as Ti_3_AlC_2_/Papyex and Ti_3_C_2_T_*x*_/Papyex, respectively. Subsequently, GNWs will be grown on the Ti_3_C_2_T_*x*_/Papyex (marked as GNWs/Ti_3_C_2_T_*x*_/Papyex). For comparison, GNWs will also be directly fabricated on the Papyex substrate (recorded as GNWs/Papyex). Figure 1 schematically illustrates the fabrication pathway for the Ti_3_C_2_T_*x*_ hybrid nanostructure. It should be noted that this architecture is not equivalent to a dense MXene film coated from only one macroscopic side. The Ti_3_C_2_T_*x*_ layer consists of delaminated and partially separated sheets with exposed basal planes, edges, and interlayer gaps. During ICP-CVD, methane-derived reactive carbon species are transported in the gas phase and can reach accessible Ti_3_C_2_T_*x*_ surfaces within the porous electrode. Therefore, GNWs can nucleate on both exposed faces of separated MXene sheets where gas-phase access is available, leading to a hierarchical GNWs/Ti_3_C_2_T_*x*_ interfacial network rather than a simple one-sided surface coating. In the electrochemical measurements, a copper substrate was employed as the current collector to ensure reliable electrical contact and accurate acquisition of the hybrid electrode’s electrochemical response.

**Figure 1:**
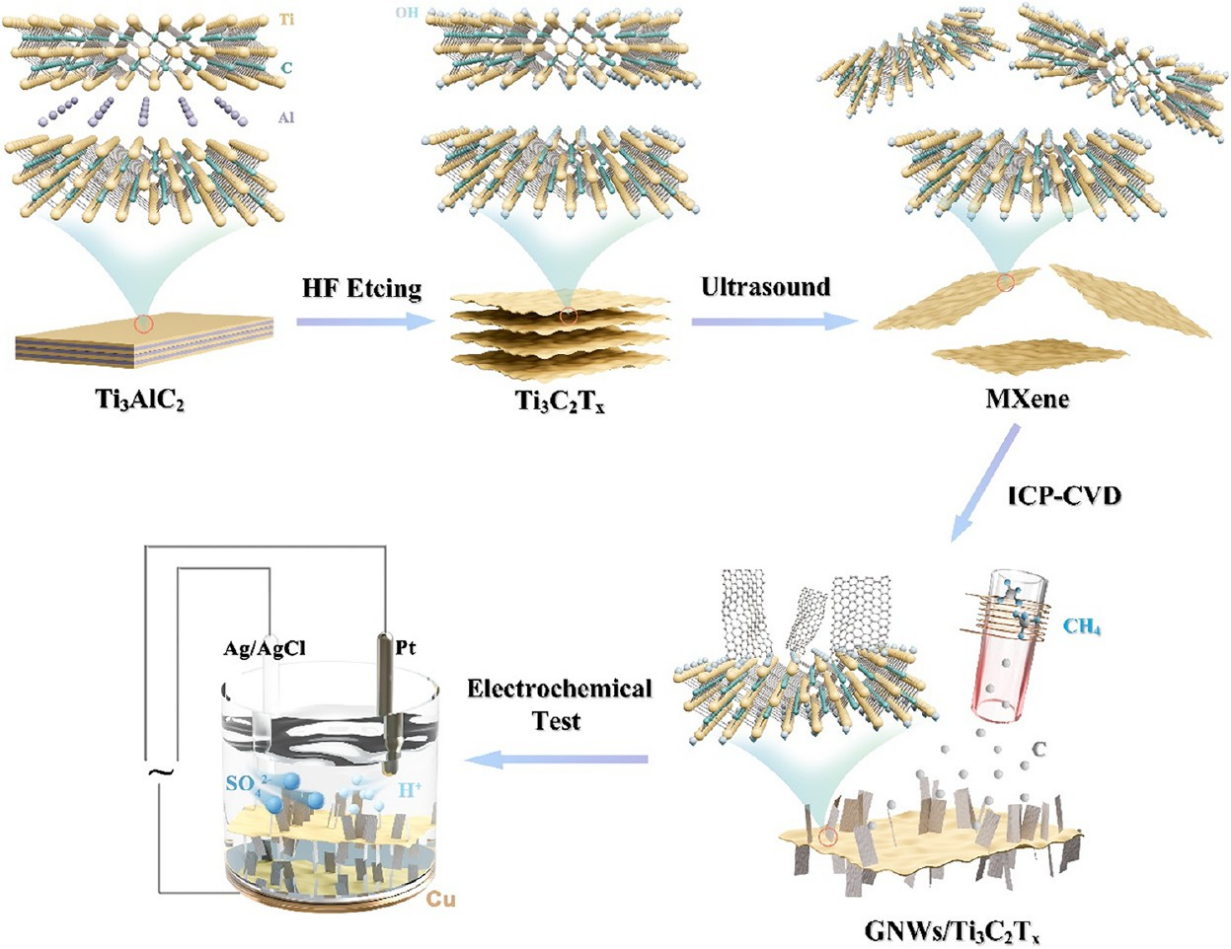
Schematic illustration of the construction of a hierarchical structure of GNWs anchored on Ti_3_C_2_T_*x*_, together with the electrochemical test based on it.

### 2.3 Material Characterization

Morphological analysis was conducted with field-emission scanning electron microscopy (FESEM; JEOL JSM-7001F) at 20 kV. High-resolution transmission electron microscopy (HRTEM, JEOL 1010) was used to investigate the microstructure of the samples at an accelerating voltage of 200 kV. The crystal structure of the GNWs/MXene composites was examined through X-ray diffraction (XRD, PANalytical X’Pert Pro MPD) employing Cu Kα radiation (50 kV) across a 2θ range of 5–90°. The wettability was assessed using a contact angle goniometer (KSV CAM200). X-ray photoelectron spectroscopy (XPS) measurements were carried out with a PHI 5500 Multi-Technique System (Physical Electronics, Chanhassen, MN, USA) equipped with a monochromatic Al Kα X-ray source.

### 2.4 Electrochemical Measurements

The electrochemical performance of the GNWs/MXene hybrid electrodes was examined in a three-electrode setup at room temperature, utilizing a 1 M aqueous H_2_SO_4_ electrolyte and an electrochemical workstation (GAMRY INSTRUMENTS, Interface 1010). The samples were used as working electrodes without further treatment. A Pt foil was used as the counter electrode, and an Ag/AgCl electrode with a 3 M KCl internal filling solution served as the reference. Cyclic voltammetry (CV) tests were performed within a potential range of −0.2 to 0.5 V, with scan rates varying from 10 to 150 mV s^–1^. Galvanostatic charge–discharge (GCD) tests were conducted at current densities from 1.5 to 3 mA cm^–2^. Electrochemical impedance spectroscopy (EIS) was performed over a frequency range of 0.1 Hz to 100 kHz at an open-circuit potential of 0.15 V, with an AC perturbation amplitude of 5 mV. The specific capacitance was determined from the GCD curves using the following equation

1 $$C_{s}=\frac{I\times t}{A\times \Delta V}$$

Where *C*_s_, *I*, *t*, *A*, and Δ*V* represent the specific areal capacitance (F cm^–2^), the charge–discharge current (A), the discharge time (s), the electrode area (cm^2^), and the discharge potential window (V), respectively. The areal specific capacitance can also be derived from CV curves using the following formula

2 $$C_{s}=\frac{1}{2v\left(V_{2}-V_{1}\right)A}\int_{V_{1}}^{V_{2}}IdV$$

Where *v*, *A*, *I*, *V*_2_, and *V*_1_ denote the scan rate (mV s^–1^), the area of the working electrode (cm^2^), the current (mA), and the upper and lower limits of the potential window (V), respectively. The areal energy density and power density are calculated using the following equations

3 $$E=\frac{1}{2}\times C_{s}\times \frac{{\left(\Delta V\right)}^{2}}{3600}$$

4 $$P=3600\times \frac{E}{t}$$

where *C*_s_, Δ*V*, and *t* represent the areal capacitance, discharge potential window, and discharge time, respectively.

## 3 Results and Discussion

As shown in Figure 2, there is a gradual structural transformation of Papyex-supported materials, starting from pristine GNWs and Ti_3_AlC_2_, progressing to delaminated Ti_3_C_2_T_*x*_, and finally forming the GNWs/Ti_3_C_2_T_*x*_ hybrid composite. As demonstrated in Figure 2a, the GNWs/Papyex specimen exhibits a highly entangled nanowall network, consisting of vertically aligned, wrinkled carbon sheets that closely envelop the substrate. This morphology provides a large, accessible surface area along with interconnected electron pathways, both of which benefit electrochemical processes. In contrast, the Ti_3_AlC_2_/Papyex surface (Figure 2b) shows the typical plate-like structure of MAX phases, consisting of large micrometer-sized grains with clearly defined cleavage planes. After removing Al from the original Ti_3_AlC_2_ compound, the resulting Ti_3_C_2_T_*x*_/Papyex (shown in Figure 2c) exhibits an accordion-like morphology, with increased interlayer spacing and greater surface roughness. The presence of these features indicates successful MXene formation, which improves hydrophilicity and facilitates subsequent GNWs growth. Figure 2d illustrates a significant morphological change in the GNWs/Ti_3_C_2_T_*x*_/Papyex composite, with dense GNWs nucleating and growing evenly over the Ti_3_C_2_T_*x*_ surface. The GNWs infiltrate the delaminated Ti_3_C_2_T_*x*_ layers, forming a hierarchical structure with vertical GNWs attached to the conductive 2D substrate. This strong interfacial bonding increases surface area and provides multidirectional electron-transfer pathways, resulting in a hybrid structure with improved mechanical and electrical properties.

**Figure 2:**
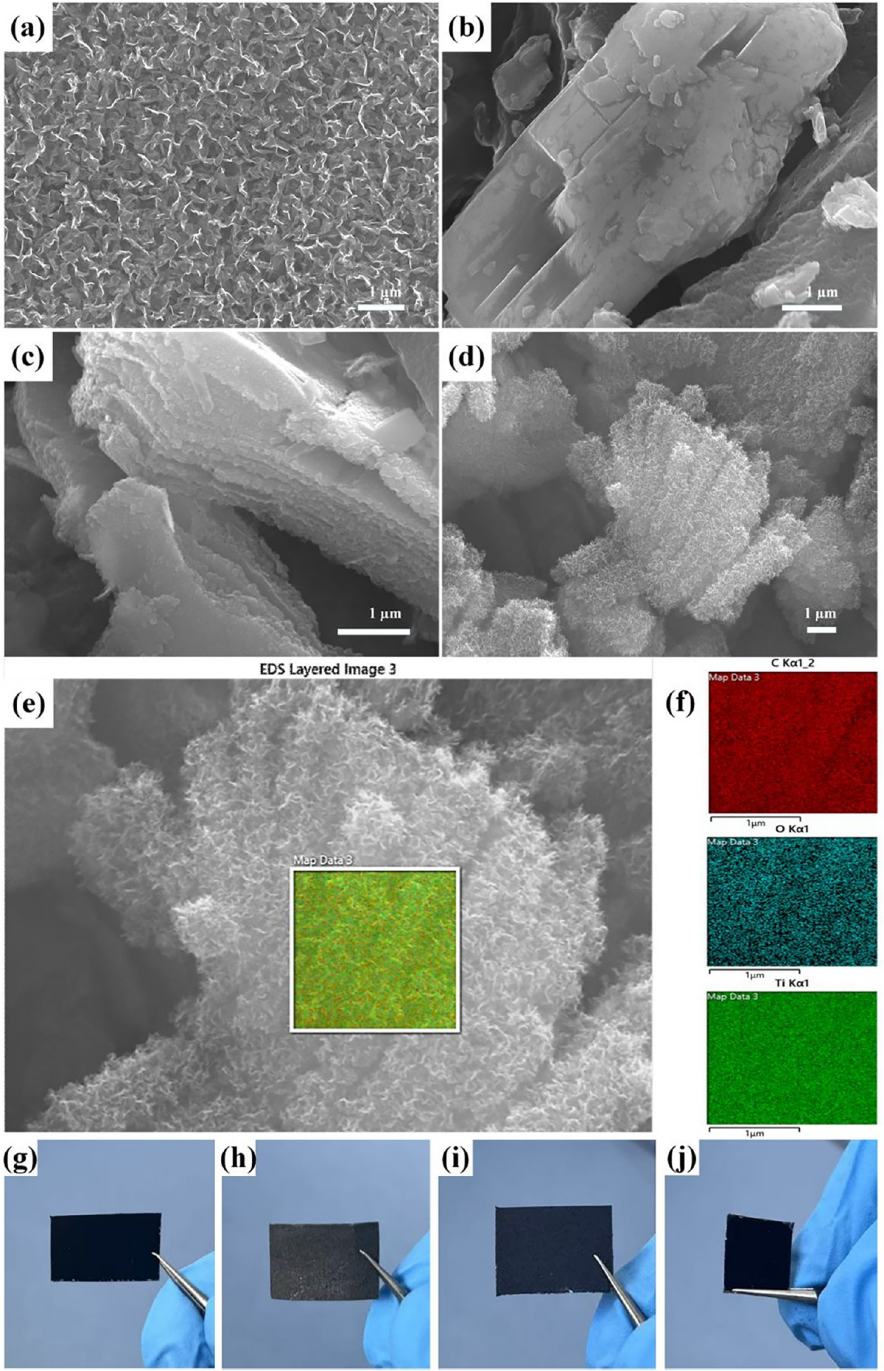
Typical top-view of (a) GNWs/Papyex, (b) Ti_3_AlC_2_/Papyex, (c) Ti_3_C_2_T_*x*_/Papyex, and (d) GNWs/Ti_3_C_2_T_*x*_/Papyex with size marking, (e) EDS spectroscopy of GNWs/Ti_3_C_2_T_*x*_/Papyex confirming GNWs on the Ti_3_C_2_T_*x*_, and elemental mapping of (f) the carbon, oxygen, and titanium on the GNWs/Ti_3_C_2_T_*x*_/Papyex sample within (g) a rectangular frame. Optical images of (g) GNWs/Papyex, (h) Ti_3_AlC_2_/Papyex, (i) Ti_3_C_2_T_*x*_/Papyex, and (j) GNWs/Ti_3_C_2_T_*x*_/Papyex.

The EDS image in Figure 2e substantiates the coexistence of GNWs and Ti_3_C_2_T_*x*_ in the composite. The homogeneous elemental distribution observed in Figure 2f further validates the successful incorporation of GNWs without disrupting the Ti_3_C_2_T_*x*_ framework. Carbon mapping has been used to highlight both the GNWs network and the underlying Ti_3_C_2_T_*x*_, while Ti mapping has reflected the MXene backbone. Finally, oxygen mapping corresponds to surface terminations formed during etching and GNWs synthesis. The uniform distribution of elements facilitates the formation of a consistent hybrid interface. Figure 2g–j provides a visual comparison of the optical material images at each processing stage. Furthermore, both GNWs/Papyex (Figure 2g) and GNWs/Ti_3_C_2_T_*x*_/Papyex (Figure 2j) display a deep black coloration, indicative of strong light absorption by nanoscale carbon structures. The intermediate Ti_3_AlC_2_/Papyex sample has a metallic gray appearance (Figure 2h), whereas the Ti_3_C_2_T_*x*_/Papyex sample (Figure 2i) adopts a darker hue, which is characteristic of delaminated MXene. These alterations align with the microstructural evolution observed using SEM.

The integration of GNWs with Ti_3_C_2_T_*x*_ produces a synergistic hybrid structure. GNWs enhance the vertical conductivity and porosity, while Ti_3_C_2_T_*x*_ contributes high in-plane conductivity, tunable surface chemistry, and mechanical support. The resulting GNWs/Ti_3_C_2_T_*x*_ architecture is therefore expected to offer significantly enhanced capacitive performance, where both high conductivity and large active surface area are critical. In practice, the growth duration of GNWs plays a decisive role in defining their architecture and therefore in regulating the trade-off between electrochemically accessible surface area and ion-transport efficiency. Figure S2 provides a systematic SEM comparison of GNWs deposited for 5, 15, 20, and 30 min, integrating top-view, 30° tilted-view, and cross-sectional observations to track the evolution from early nucleation to a mature porous network. After 5 min of GNWs growth (Figure S2a–a2), large regions of the Papyex substrate remain exposed and are decorated only by sparsely distributed nanosheet fragments, indicative of a nucleation-limited regime. The discontinuous coverage and low wall density suggest preferential nucleation at high-energy sites, limited lateral expansion, and insufficient vertical development to establish an interconnected conductive framework. Such a morphology is expected to offer a relatively low density of edge-rich electroactive sites and an incomplete electron-transport network. By 15 min (Figure S2b–b2), the surface transitions into a continuous GNWs “carpet” composed of densely packed, vertically oriented walls. The higher edge density and reduced bare-substrate exposure observed in the top-view image, together with the tilted and cross-sectional views, confirm the formation of a conformal porous layer with comparatively uniform thickness. This architecture is advantageous for charge storage because abundant edges and defects provide adsorption and charge-accumulation sites, while open interwall voids facilitate electrolyte infiltration and shorten ion-diffusion pathways. At 20 min (Figure S2c–c2), the GNWs network exhibits enhanced structural integration. The walls appear more interwoven and homogeneous, and the cross-section reveals an increased thickness relative to 15 min without evidence of a compact, sealing overlayer. The persistence of a rough, porous upper region implies sustained vertical growth and continued electrolyte accessibility, supporting rapid charge transport through the conductive scaffold while preserving ion-accessible porosity, which typically underpins high capacitance and good rate capability. Extending the growth to 30 min (Figure S2d–d2) further develops the GNWs layer; however, the incremental thickness increase becomes less pronounced, consistent with an approach toward a saturation-like state. Simultaneously, the tilted and cross-sectional images indicate local coarsening and partial aggregation near the top surface, consistent with the onset of secondary lateral thickening and wall–wall interactions.^40^ Under these conditions, deposition increasingly reinforces existing walls and bridges rather than creating new open channels, thereby elevating tortuosity and potentially constricting interwall spacing. Continued densification beyond this stage is expected to hinder electrolyte penetration and slow ion transport, thereby reducing the effectively utilized surface area under practical scan rates and current densities.

Considering these trends, a growth time of 30 min was selected as the optimal condition for this study. At this duration, the GNWs layer approaches a thickness plateau (∼1 μm) while maintaining a robust conductive framework and an open nanowall topology. To further support growth-time optimization, CV measurements were performed on GNWs/Papyex electrodes with varying growth durations. As shown in Figure S3, the enclosed CV area increases progressively from 5 to 30 min at a scan rate of 10 mV s^–1^. The GNWs/Papyex-5 min sample exhibits the weakest capacitive response, consistent with incomplete GNWs coverage and insufficient formation of interconnected conductive pathways. With increasing growth time, the CV area increases, indicating improved charge-storage capability due to higher nanowall density and a more accessible electroactive surface. Among the tested conditions, the GNWs/Papyex-30 min electrode exhibits the largest CV area, indicating that this growth duration yields the most favorable electrochemical response. Combined with the SEM results in Figure S2, this CV comparison supports the selection of 30 min as the optimized GNWs growth condition for constructing the GNWs/Ti_3_C_2_T_*x*_/Papyex hybrid electrode. Further prolonging the growth is likely to promote lateral overgrowth and top convergence, which would obstruct ion-diffusion pathways and diminish the available surface for ion adsorption, ultimately compromising charge-storage efficiency. The textural characteristics of this Ti_3_C_2_T_*x*_-based material system were previously investigated in detail by N_2_ adsorption–desorption measurements.^47^ The BET surface area increased from 34.37 m^2^ g^–1^ for Ti_3_C_2_T_*x*_ to 45.99 m^2^ g^–1^ for GNWs/Ti_3_C_2_T_*x*_, while the total pore volume increased from 0.156 to 0.209 cm^3^ g^–1^ after GNWs incorporation. The average pore size remained in the mesoporous range, approximately 18.19 nm for Ti_3_C_2_T_*x*_ and 18.17 nm for GNWs/Ti_3_C_2_T_*x*_. The corresponding Type IV N_2_ adsorption–desorption isotherms further confirmed the mesoporous nature of the hybrid architecture. These textural parameters support the electrochemical results obtained in the present work, where GNWs/Ti_3_C_2_T_*x*_/Papyex exhibits the highest areal capacitance. The enhanced performance is attributed not only to the increased surface area and pore volume but also to the GNWs-induced suppression of MXene restacking, improved electrolyte accessibility, and more efficient utilization of redox-active Ti_3_C_2_T_*x*_ surface terminations.

The structural evolution from Ti_3_AlC_2_ to Ti_3_C_2_T_*x*_, and subsequently to the GNWs/Ti_3_C_2_T_*x*_ hybrid, is clearly evidenced by the XRD patterns in Figure 3a. The GNWs/Papyex sample demonstrates a dominant reflection at 2θ ≈ 26°, corresponding to the graphitic (002) plane (JCPDS: 00–001–0640). Together with the weaker (004) reflection, this feature is consistent with turbostratic graphitic stacking, as typically observed for vertical GNWs grown on carbon substrates. In contrast, pristine Ti_3_AlC_2_/Papyex displays a series of sharp, high-intensity peaks indexed to the (002), (004), (011), (012), (013), (015), (018), and higher-order reflections, confirming the highly crystalline, well-ordered hexagonal MAX-phase structure. After selective Al removal, Ti_3_C_2_T_*x*_/Papyex shows pronounced structural changes: the (002) and (004) reflections shift toward lower angles and broaden substantially, indicating expansion of the interlayer spacing and increased structural disorder—hallmarks of successful MXene formation.^48^ The interlayer spacing was further calculated from the low-angle (002) reflection using Bragg’s law with λ = 1.54 Å, where λ is the wavelength of Cu Kα radiation. The (002) peak of Ti_3_AlC_2_ appears at approximately 9.5°, corresponding to an interlayer spacing of 0.93 nm. After selective Al etching, the (002) reflection of Ti_3_C_2_T_*x*_/Papyex shifts to a lower angle of approximately 8.9°, giving an enlarged spacing of 1.00 nm. This increase confirms the expansion of the layered structure after MAX-to-MXene conversion, which is associated with Al removal and the introduction of surface terminations/intercalated species. For GNWs/Ti_3_C_2_T_*x*_/Papyex, the broadened low-angle MXene-related peak (∼8.9°) corresponds to a similar interlayer spacing (∼1.00 nm) compared to Ti_3_C_2_T_*x*_/Papyex, indicating that the expanded Ti_3_C_2_T_*x*_ framework is maintained after GNWs growth. The enlarged interlayer spacing is favorable for electrolyte access and ion diffusion, helping to explain the enhanced capacitance of the GNWs/Ti_3_C_2_T_*x*_/Papyex electrode. At the same time, the marked attenuation/disappearance of characteristic Ti_3_AlC_2_ reflections (e.g., (008) and (012)) evidence loss of long-range MAX-phase order and supports the conversion to delaminated Ti_3_C_2_T_*x*_. Following GNWs deposition, the GNWs/Ti_3_C_2_T_*x*_/Papyex composite retains the broadened MXene (002) feature while exhibiting enhanced carbon-related intensity near 26°, attributable to GNWs anchored on the Ti_3_C_2_T_*x*_ surface. The coexistence of MXene and graphitic reflections confirms effective hybridization. In addition, partial masking of higher-angle MXene peaks by the carbon contribution is consistent with a conformal GNWs overlayer on Ti_3_C_2_T_*x*_, yielding a hierarchical architecture that couples Ti_3_C_2_T_*x*_ sheets with a 3D GNWs nanonetwork. The observed XRD evolution aligns with the intended hierarchical design. The expanded interlayer spacing of Ti_3_C_2_T_*x*_ enhances ion intercalation and provides abundant active sites, while GNWs provide mechanical reinforcement, increased conductivity, and additional accessible surface area. The combination of delaminated Ti_3_C_2_T_*x*_ with vertical GNWs creates multidimensional electron-transport pathways and enhances structural robustness.

**Figure 3:**
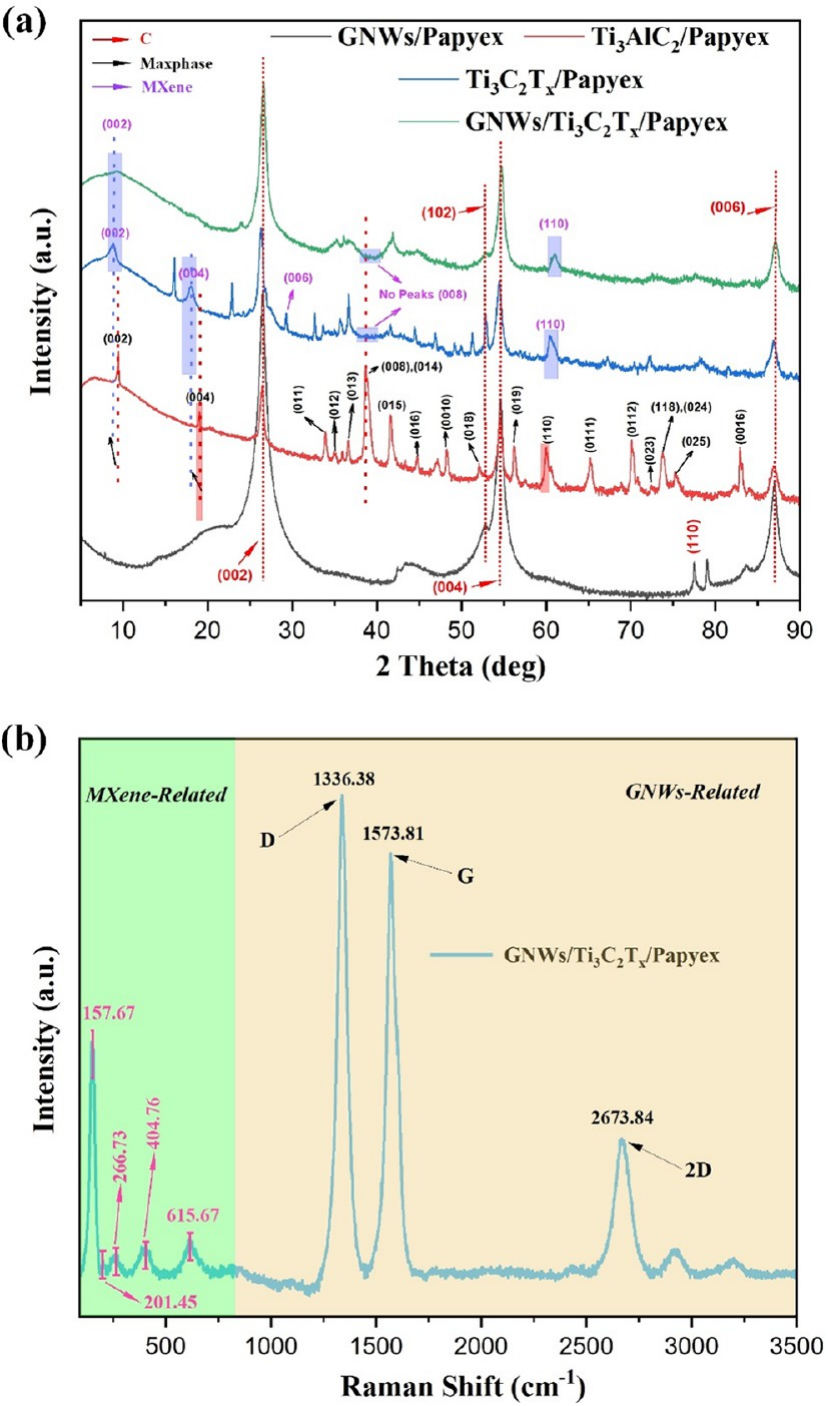
XRD patterns (a) of GNWs/Papyex, Ti_3_AlC_2_/Papyex, Ti_3_C_2_T_*x*_/Papyex, and GNWs/Ti_3_C_2_T_*x*_/Papyex; Raman spectrum (b) of GNWs/Ti_3_C_2_T_*x*_/Papyex.

Raman spectroscopy further corroborates these structural features (shown in Figure 3b). The spectrum can be divided into two characteristic regions, namely MXene lattice vibrations (≈150–700 cm^–1^) and graphitic carbon modes from GNWs (≈1200–3000 cm^–1^), and their coexistence provides direct evidence of successful integration on the Papyex substrate. In the low-wavenumber range, well-defined bands at 157.67, 201.45, 266.73, 404.76, and 615.67 cm^–1^ are characteristic of Ti_3_C_2_T_*x*_ and are commonly associated with Ti–C vibrations and termination-related modes (Ti-T_*x*_; T_*x*_ typically includes –O/–OH/–F) formed during HF etching.^49^ In the high-wavenumber region, the pronounced D band at 1336.38 cm^–1^ reflects disorder-activated scattering in sp^2^ carbon and is consistent with the edge- and defect-rich nature of vertical GNWs. The G band at 1573.81 cm^–1^, corresponding to the E_2g_ in-plane stretching mode of graphitic carbon, confirms the presence of ordered sp^2^ domains within the nanowalls.^50^ Its slight shift relative to pristine graphite implies interfacial effects such as strain or charge transfer between GNWs and Ti_3_C_2_T_*x*_. The 2D band at 2673.84 cm^–1^ appears broad and low in intensity, characteristic of few- or multilayer turbostratic graphene, as expected for GNWs. The concurrent presence of preserved MXene vibrational modes and strong graphitic features confirms that the GNWs/Ti_3_C_2_T_*x*_ hybrid maintains the structural integrity of both components, while subtle shifts and broadening suggest interfacial interactions that may promote electron transport and reduce charge-transfer resistance.

Figure S4 enables a comparative assessment of the carbon-framework Raman signatures and Ti–C-related vibrational features of GNWs/Papyex, Ti_3_AlC_2_/Papyex, and Ti_3_C_2_T_*x*_/Papyex. In the 800–3500 cm^–1^ window (Figure S4a), all samples exhibit the characteristic carbon D band (≈1351 cm^–1^) and G band (≈1587 cm^–1^), originating primarily from the Papyex carbon support, with additional contributions from GNWs where present. The extracted defect parameters follow *I*_D_/*I*_G_ = 1.16 for GNWs/Papyex, 0.89 for Ti_3_AlC_2_/Papyex, and 0.75 for Ti_3_C_2_T_*x*_/Papyex. The higher *I*_D_/*I*_G_ ratio of GNWs/Papyex is compatible with the defect-rich nature of vertical GNWs,^51^ where abundant boundaries and curvature enhance disorder-activated scattering. By contrast, the lower *I*_D_/*I*_G_ for Ti_3_C_2_T_*x*_/Papyex suggests a relatively larger contribution from ordered sp^2^ carbon environments, largely dominated by the Papyex substrate. A 2D band at around 2700 cm^–1^ is clearly visible for GNWs/Papyex, confirming the existence of graphitic domains. Its broadened profile is characteristic of few-layer graphene with turbostratic stacking and nonuniform thickness. The low-wavenumber region (100–800 cm^–1^, shown in Figure S4b) reveals vibrational modes associated with the Ti–C framework and termination-sensitive features in MXene. Ti_3_AlC_2_/Papyex displays distinct bands at approximately 195.9 cm^–1^ and 270.3 cm^–1^, which are assigned to an E_2g_-type vibration involving Ti/Al/C and an A_1g_-type mode related to Ti–C bonding, respectively, in agreement with the established Raman response of the Ti_3_AlC_2_ MAX phase.^52^ Upon etching and Al removal, the corresponding A_1g_-related feature in Ti_3_C_2_T_*x*_ is expected to shift to a lower wavenumber, reflecting the altered lattice dynamics. In line with the formation of MXenes, Ti_3_C_2_T_*x*_/Papyex presents broadened, termination-sensitive bands in the so-called T_*x*_ region, commonly associated with Ti–C coupled vibrations modulated by surface terminations (e.g., –O/–OH), together with a higher-wavenumber “C region” contribution arising from the carbon lattice. By comparison, GNWs/Papyex remains essentially featureless in the 100–800 cm^–1^ range, as expected for predominantly graphitic carbon whose strongest Raman features reside in the D, G, and 2D bands rather than at low wavenumbers.

The microstructure of the GNWs/Ti_3_C_2_T_*x*_ hybrid was investigated by TEM (Figure 4). The low-magnification image (Figure 4a) shows a representative Ti_3_C_2_T_*x*_ flake with a relatively dense interior that is surrounded by a lighter, porous shell composed of GNWs. The GNWs conformally cover the Ti_3_C_2_T_*x*_ surface and protrude outward, creating a core–shell configuration. At higher magnification (Figure 4b), the GNWs are observed to be directly anchored on the surface of the Ti_3_C_2_T_*x*_ nanosheet, generating a rough and corrugated interfacial region. The stability of the GNWs/Ti_3_C_2_T_*x*_ hybrid is closely related to the interfacial coupling between the two components. Because GNWs are grown in situ on Ti_3_C_2_T_*x*_/Papyex by ICP-CVD, the interface is formed through direct nucleation and anchoring of carbon nanowalls on accessible Ti_3_C_2_T_*x*_ surfaces rather than through simple physical mixing. The surface terminations of Ti_3_C_2_T_*x*_, including –O, −OH, and –F groups, can interact with edge/defect sites and oxygen-containing groups on GNWs through polar interactions, hydrogen bonding, and van der Waals contact. In addition, interfacial electronic coupling between Ti_3_C_2_T_*x*_ and graphitic carbon can facilitate charge redistribution and electron transport across the hybrid interface. This coupled interface allows GNWs to act as conductive spacers that suppress Ti_3_C_2_T_*x*_ restacking, stabilize the open layered framework, and maintain efficient ion/electron transport during cycling. High-resolution TEM (HRTEM) provides further insight into the crystalline characteristics of both components. The HRTEM image focusing on the GNWs (Figure 4c) shows well-resolved lattice fringes, signifying a high degree of graphitic ordering. This is supported by the corresponding FFT pattern (Figure 4d), which contains distinct diffraction spots characteristic of graphitic carbon. From the enlarged HRTEM view (Figure 4e), an interplanar spacing of approximately 0.346 nm is measured and assigned to the graphene (002) planes. Line-profile analysis (Figure 4f), acquired over ∼ 2.423 nm across seven fringes, illustrates a nearly periodic intensity modulation, consistent with the measured spacing. The Ti_3_C_2_T_*x*_ domains are highlighted in Figure 4g, with extended parallel fringes across a broad region confirming the layered nature of the MXene sheets. The FFT pattern shown in Figure 4h reveals diffraction spots that can be indexed to the Ti-based MXene phase, demonstrating that the crystalline framework of Ti_3_C_2_T_*x*_ remains intact following the growth of GNWs. Closer inspection of the Ti_3_C_2_T_*x*_ lattice (Figure 4i) yields an interplanar spacing of ∼0.263 nm, aligned with the (0110) plane of Ti_3_C_2_T_*x*_. The corresponding line profile (Figure 4j), extracted over ∼2.945 nm across 15 fringes, exhibits regular periodicity, which implies good crystallinity and structural uniformity within the MXene domains. The TEM analysis confirms the successful construction of a tightly coupled GNWs/Ti_3_C_2_T_*x*_ hybrid. This synergistic nanoarchitecture is expected to shorten electron/ion transport pathways, reduce internal resistance, and thereby underpin the enhanced capacitive performance discussed in subsequent sections.

**Figure 4:**
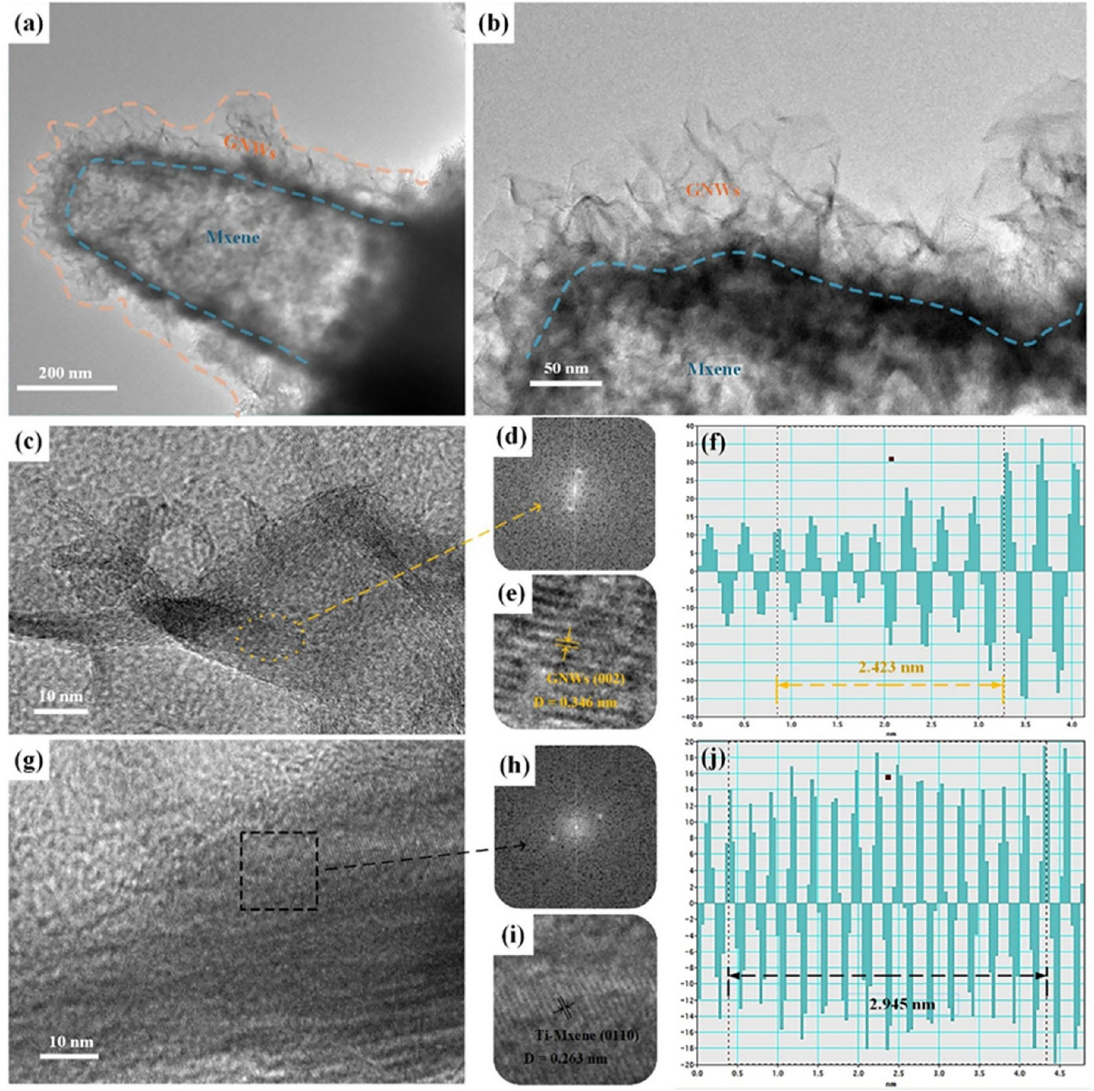
(a) Low- and (b) high-magnification TEM images showing GNWs grown on the surface of Ti_3_C_2_T_*x*_; HRTEM images of the (c) GNWs and (g) Ti_3_C_2_T_*x*_ regions; corresponding FFT patterns confirming the crystalline nature of (d) GNWs and (h) Ti_3_C_2_T_*x*_ phases; enlarged lattice fringes of (e) GNWs and (i) Ti_3_C_2_T_*x*_ with interplanar spacings; line-profile analyses of the (f) GNWs and (j) Ti_3_C_2_T_*x*_.

The XPS survey spectra of Ti_3_C_2_T_*x*_/Papyex and GNWs/Ti_3_C_2_T_*x*_/Papyex (Figure S5) display prominent C 1s, O 1s, F 1s, and Ti 2p signals along with the associated Auger features. It can be found that high intensities of O and F are consistent with the introduction of surface terminations (–O, −OH, and –F) during etching and delamination. Especially for GNWs/Ti_3_C_2_T_*x*_/Papyex, the survey spectrum is dominated by the C 1s signal, while the Ti 2p features are significantly weaker, due to GNWs’ coverage of the exposed Ti_3_C_2_T_*x*_ surface. Owing to the inherently surface-sensitive nature of XPS (information depth of only a few nanometers), the GNWs overlayer effectively screens the underlying Ti_3_C_2_T_*x*_, so that the detected photoelectrons arise predominantly from the outer carbon layer rather than the buried Ti_3_C_2_T_*x*_. The chemical composition and surface bonding states of the GNWs/Ti_3_C_2_T_*x*_ hybrids are shown in Figure 5. The high-resolution C 1s spectrum of GNWs/Ti_3_C_2_T_*x*_/Papyex (Figure 5a) is dominated by a component at ∼284.80 eV assigned to sp^2^ C–C, evidencing a highly graphitized and electronically conductive GNWs coating. Minor contributions at around 285.80 and 288.72 eV are attributed to oxygen-containing carbon species (e.g., C–O and more strongly oxidized C=O) associated with defect/edge sites of GNWs.^43^ It is noteworthy that the absence of a distinguishable Ti–C component in the C 1s spectrum further suggests that the response is governed by the GNWs overlayer, with the underlying MXene contribution significantly reduced due to overlayer screening. This surface-screening effect should be considered when correlating XPS results with the pseudocapacitive mechanism of the hybrid electrode. Because XPS probes only the outermost few nanometers, the spectrum of GNWs/Ti_3_C_2_T_*x*_/Papyex mainly reflects the graphitized GNWs overlayer rather than the buried Ti_3_C_2_T_*x*_ surface. Therefore, the directly exposed Ti_3_C_2_T_*x*_/Papyex electrode provides a more reliable reference for identifying Ti_3_C_2_T_*x*_ surface terminations. In this context, the GNWs/Ti_3_C_2_T_*x*_/Papyex XPS spectrum confirms the formation of a conductive GNWs coating, whereas the Ti_3_C_2_T_*x*_/Papyex spectra are used to evaluate the Ti-related oxygen-containing surface environments that may contribute to pseudocapacitive charge storage in an acidic electrolyte.

**Figure 5:**
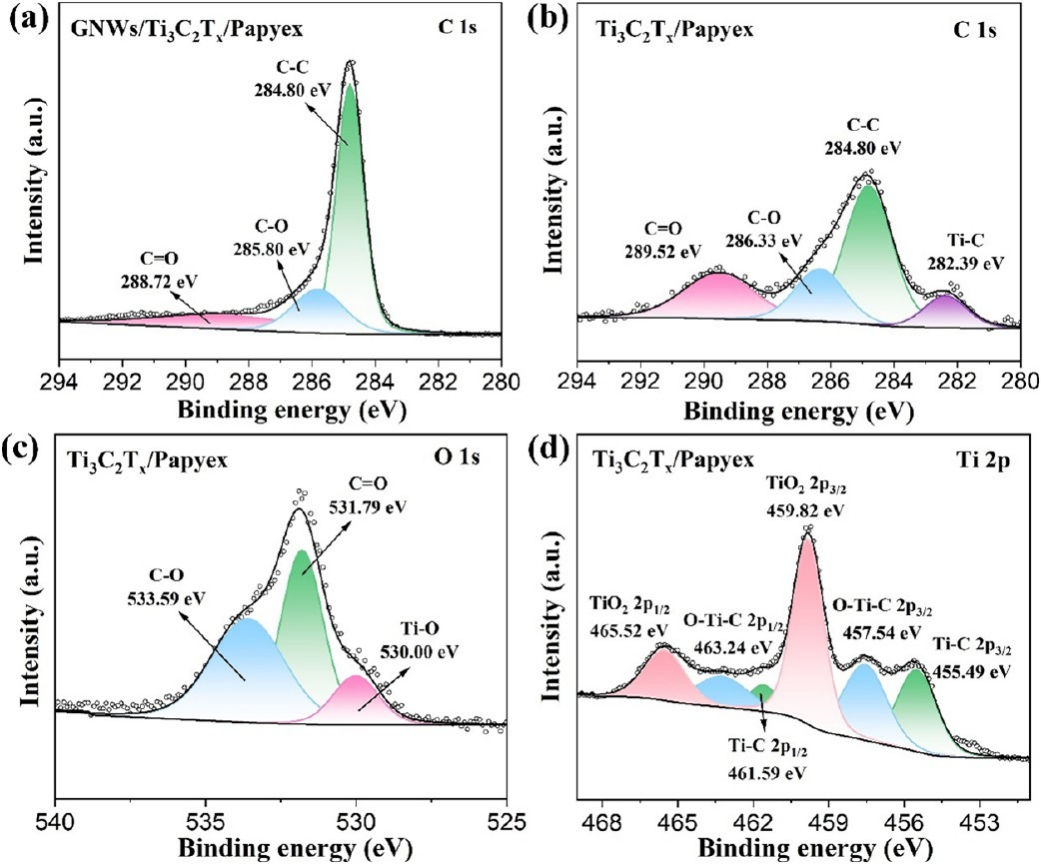
High-resolution (a) C 1s of GNWs/Ti_3_C_2_T_*x*_/Papyex; high-resolution (b) C 1s, (c) O 1s, and (d) Ti 2p spectra of the Ti_3_C_2_T_*x*_/Papyex.

To elucidate the bonding environments of C, O, and Ti in the directly exposed MXene, the Ti_3_C_2_T_*x*_/Papyex sample was analyzed in detail. The high-resolution C 1s spectrum of Ti_3_C_2_T_*x*_/Papyex (Figure 5b) can be deconvoluted into four distinct components, confirming the existence of the MXene carbide framework and oxygenated surface species. The low-binding-energy feature at about 282.39 eV is assigned to Ti–C bonding, providing direct evidence of the Ti_3_C_2_T_*x*_ carbide structure. The dominant contribution at ∼284.80 eV corresponds to C–C species, which comes from the carbon source in the Ti–C bond. The oxygen-containing carbon environments are captured by the component at ∼286.33 eV, assigned to C–O, while the higher-binding-energy peak at ∼289.52 eV is ascribed to C=O groups. The O 1s spectrum (Figure 5c) can be fitted with three components at approximately 530.00, 531.79, and 533.59 eV. The low-binding-energy peak at 530.00 eV is assigned to Ti–O species, reflecting partial surface oxidation (TiO_*x*_/TiO_2_) during processing or air exposure.^23,52^ The higher-binding-energy features at 531.79 and 533.59 eV are associated with oxygenated environments at MXene terminations and adsorbed species (e.g., C=O and C–O). Such oxygen functionalities (–O/–OH) are known to enhance hydrophilicity and can facilitate rapid surface redox reactions. The Ti 2p spectrum of Ti_3_C_2_T_*x*_/Papyex (Figure 5d) further confirms the coexistence of multiple Ti chemical states. The component at around 455.49 eV (Ti 2p_3/2_) is characteristic of Ti–C bonding within the Ti_3_C_2_T_*x*_ lattice, verifying that the carbide framework is retained after etching. Peaks at approximately 457.54 eV (Ti 2p_3/2_) and 463.24 eV (Ti 2p_1/2_) are assigned to O–Ti–C species, in line with partially oxidized Ti associated with surface terminations.^53^ The higher-binding-energy doublet at ∼459.82 and ∼465.52 eV corresponds to Ti^4+^ in TiO_2_, evidencing the presence of a thin oxide layer. This combination of Ti–C, O–Ti–C, and TiO_2_ is typical for Ti_3_C_2_T_*x*_ and reflects a surface enriched in chemically active Ti environments. The XPS results demonstrate that (1) HF etching of Ti_3_AlC_2_ produces Ti_3_C_2_T_*x*_ with abundant –O/–OH containing terminations and partially oxidized Ti species, and (2) subsequent ICP-CVD growth yields a continuous, graphitized GNWs overlayer that effectively masks the MXene signal in the GNWs/Ti_3_C_2_T_*x*_/Papyex spectra.

In contrast, as shown in Figure S6, the survey (Figure S6a) and high-resolution XPS spectra (Figure S6b–d) of Ti_3_AlC_2_/Papyex confirm MAX-phase carbide bonding, with certain contributions from oxygen-containing species. In the C 1s region, the dominant component at around 284.80 eV is assigned to C–C bonding, whereas the low-binding-energy feature at ∼282.15 eV corresponds to Ti–C, confirming the carbide framework of Ti_3_AlC_2_. Additional contributions at about 286.08 eV (C–O) and 289.88 eV (C=O) indicate a small fraction of oxygenated carbon functionalities associated with surface groups or ambient exposure. The O 1s spectrum further distinguishes these oxygen environments, showing a component at ∼531.10 eV linked to metal-O bonds. Additionally, there are peaks at around 532.41 eV (C=O) and 533.83 eV (C–O), which are indicative of hydroxyl or ether-like species. The Ti 2p spectrum displays the characteristic Ti–C doublet at ∼455.08 eV (Ti 2p_3/2_) and ∼461.58 eV (Ti 2p_1/2_), in conjunction with supplementary features at about 456.92/462.72 eV, which are attributed to O–Ti–C species arising from partial surface oxidation.^54^ A higher-binding-energy doublet of TiO_2_ (Ti^4+^) at ∼459.57/∼465.27 eV is also observed, showcasing that oxidation is confined to a thin surface layer.

Prior to electrochemical testing, it is important to certify that the hybrid electrodes exhibit sufficient hydrophilicity because improved wettability enhances electrolyte-electrode contact, promotes ion transport, and ultimately benefits electrochemical performance. Figure S7 compares the wetting behavior of pristine Papyex, Ti_3_AlC_2_/Papyex, Ti_3_C_2_T_*x*_/Papyex, and the GNWs/Ti_3_C_2_T_*x*_/Papyex hybrid. Pristine Papyex (Figure S7a–a1) shows a moderate contact angle of ∼59.0° (61.18° left/56.73° right), in accordance with the relatively low surface energy of graphitic carbon paper. After coating with Ti_3_AlC_2_ (Figure S7b–b1), the contact angle increases to ∼81.0° (80.84°/81.24°), indicating a more hydrophobic surface, which agrees with the lower density of polar surface functionalities characteristic of the MAX phase. Converting Ti_3_AlC_2_ to Ti_3_C_2_T_*x*_ by selective etching (Figure S7c–c1) decreases the contact angle to ∼71.6° (73.00°/70.15°), reflecting enhanced wettability attributable to the formation of hydrophilic surface terminations (–O/–OH). A markedly different wetting regime is observed for GNWs/Ti_3_C_2_T_*x*_/Papyex (Figure S7d–d1). It has been demonstrated that a stable droplet is unable to be sustained; instead, the liquid rapidly disperses and penetrates into the porous architecture, corresponding to essentially complete wetting (θ ≈ 0°). This strong electrolyte affinity is expected to reduce wetting-related interfacial resistance, improve electrolyte infiltration, and facilitate rapid ion access to internal active surfaces.

The prepared GNWs/Ti_3_C_2_T_*x*_ hybrid displays a distinct layered structure and numerous functional groups, making it suitable for Faraday redox reactions. As a result, it has the potential to function as an electrode material in supercapacitors. Our research systematically examined the GCD and CV profiles of GNWs/Papyex, Ti_3_AlC_2_/Papyex, Ti_3_C_2_T_*x*_/Papyex, and GNWs/Ti_3_C_2_T_*x*_/Papyex using a 1 M H_2_SO_4_ electrolyte. The 1 M H_2_SO_4_ electrolyte offers adequate ionic conductivity for effective electrochemical reactions while ensuring a controlled experimental setting. Additionally, it facilitates appropriate Faraday reaction rates for redox processes and remains highly compatible with MXene during these reactions. In Figure S8, CV curves for GNWs/Papyex, Ti_3_AlC_2_/Papyex, Ti_3_C_2_T_*x*_/Papyex, and GNWs/Ti_3_C_2_T_*x*_/Papyex hybrids are presented at various scan rates. The absence of symmetry in the electrode curves is presumably attributable to the combination of EDLC and pseudocapacitance, which collectively contribute to the overall capacitance. Figure 6a shows that the GNWs/Ti_3_C_2_T_*x*_/Papyex has the largest CV curve area at a scan rate of 100 mV s^–1^ among the electrodes, demonstrating its superior charge storage capacity. As illustrated in Figure 6b, the GCD curves of these configurations are shown at a current density of 1.5 mA cm^–2^. The GCD profile of GNWs/Ti_3_C_2_T_*x*_/Papyex deviates slightly from an ideal triangular shape, reflecting the coexistence of EDLC and pseudocapacitive charge-storage processes, in agreement with the CV analysis. The charge and discharge durations of this electrode are comparable, at approximately 40 and 41 s, respectively. The corresponding Coulombic efficiency is about 102.5%, close to 100%, indicating largely reversible charge storage. The slightly longer discharge time can be attributed to the mixed EDLC/pseudocapacitive behavior of the hybrid electrode, together with ion redistribution and transient polarization within the hierarchical porous structure. Importantly, GNWs/Ti_3_C_2_T_*x*_/Papyex still exhibits the longest discharge duration among the tested electrodes under the same current density, confirming its higher charge-storage capability. The apparent voltage drop at the charge–discharge transition was considered as well. In GCD measurements, this voltage drop is related to internal resistance and transient polarization, and it generally decreases when the applied current density is reduced. In Figure 6b, GNWs/Ti_3_C_2_T_*x*_/Papyex does not show the smallest voltage drop, which may be associated with polarization within its hierarchical porous structure and pseudocapacitive charge-storage process. Nevertheless, the hybrid electrode exhibits the longest discharge time under the same current density, confirming its higher charge-storage capability among the tested samples. Specifically, Figure 6c compares the specific capacitance calculated from CV curves for all samples tested at varying scan rates using eq 2. The capacitances for GNWs/Papyex, Ti_3_AlC_2_/Papyex, and Ti_3_C_2_T_*x*_/Papyex are 22.5, 39.9, and 133.5 mF cm^–2^, respectively, at a scan rate of 10 mV s^–1^. It can be seen that the GNWs/Ti_3_C_2_T_*x*_/Papyex demonstrates the highest capacitance, attaining an impressive value of 163.2 mF cm^–2^, which is 7.3 times greater than that of GNWs/Papyex and 1.2 times greater than that of Ti_3_C_2_T_*x*_/Papyex. The decline in CV capacitance consistently increases with higher scan rate, which probably results from limited ion penetration into the nanopore entrances due to rapid potential shifts. Figure S9 exhibits the GCD curves of these four samples at current densities of 1.5–3 mA cm^–2^. The enhanced performance of GNWs/Ti_3_C_2_T_*x*_/Papyex can be attributed to the intercalation of GNWs, which prevents MXene restacking and self-aggregation, and creates multidirectional, stable ion-transport channels.

**Figure 6:**
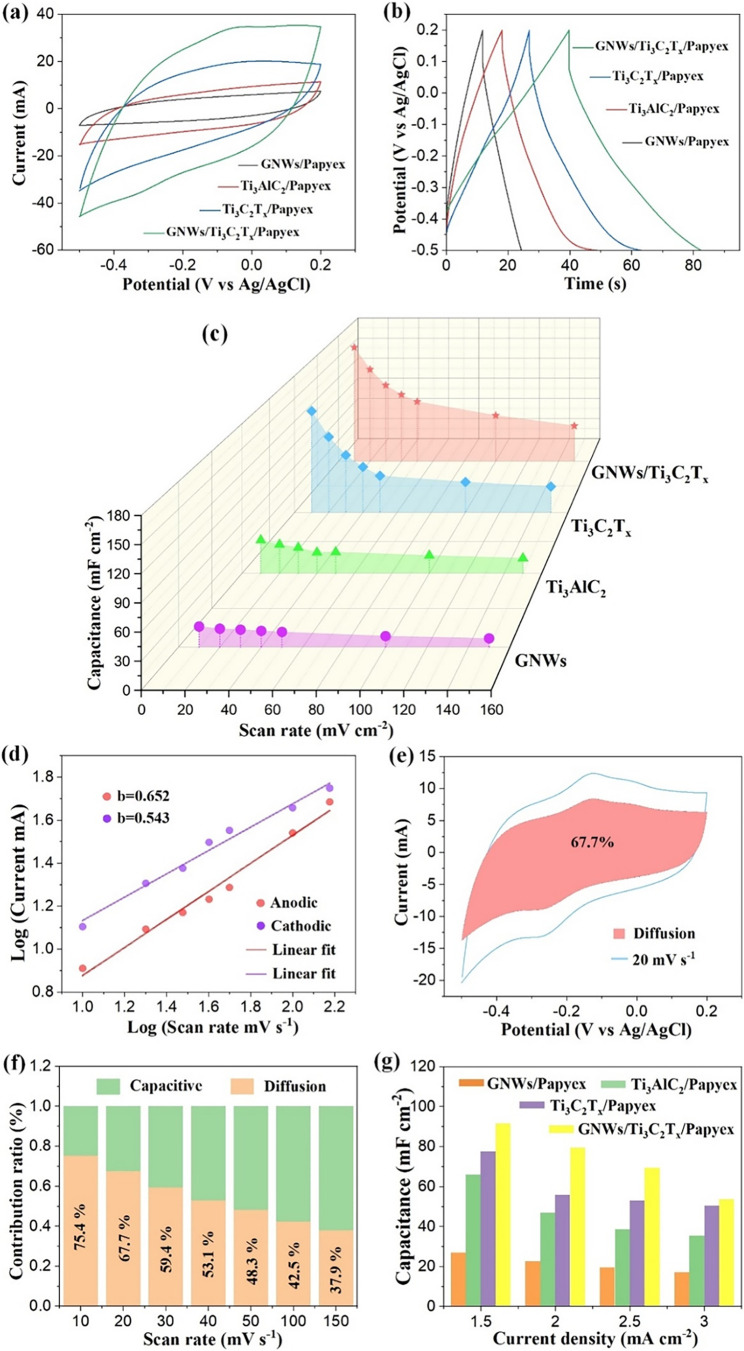
(a) CV curves of GNWs/Papyex, Ti_3_AlC_2_/Papyex, Ti_3_C_2_T_*x*_/Papyex, and GNWs/Ti_3_C_2_T_*x*_/Papyex at a scan rate of 100 mV s^–1^; (b) GCD curves of GNWs/Papyex, Ti_3_AlC_2_/Papyex, Ti_3_C_2_T_*x*_/Papyex, and GNWs/Ti_3_C_2_T_*x*_/Papyex at the current density of 1.5 mA cm^–2^; (c) specific areal capacitances of all the samples at different scan rate calculated from CV curves; (d) log­(*i*) versus log­(*v*) plots of the current peaks at different scan rates, (e) diffusion contribution, and (f) the proportion of normalized diffusion contribution of the GNWs/Ti_3_C_2_T_*x*_/Papyex at each scan rate; (g) specific areal capacitances shown in 2D column at various current densities for all the samples.

The study of response dynamics, focusing on comparing peak current (*i*) and scan rate (ν), was performed by analyzing the following equations

5 $$i=av^{b}$$

6 log(i)=log(a)+b⁡log(v)

As shown in eqs 5 and 6, *a* and *b* are constants. In particular, the parameter *b*, ranging from 0.5 to 1.0, plays an instrumental role in evaluating the charge storage mechanism. The gradient of the log­(*i*) versus log­(*v*) plot is an indication of the process. In a semi-infinite linear system, the capacitance contribution is diffusion-controlled as *b* nears 0.5, and surface-controlled when *b* converges to 1.0.^55^ Notably, the GNWs/Ti_3_C_2_T_*x*_/Papyex electrode shows *b* values of 0.543 at the cathodic peak and 0.652 at the anodic peak (Figure 6d). The relatively low *b*-values of GNWs/Ti_3_C_2_T_*x*_/Papyex indicate that the charge-storage process is not purely surface-controlled, but contains a substantial diffusion-assisted contribution. This behavior differs from highly surface-dominated MXene systems reported in the literature, such as porous MXene/carbon-dot films with 3D interconnected ion-transport channels and LTMS-derived V_4_C_3_T_*x*_ electrodes with expanded interlayer spacing.^56,57^ In the present Papyex-supported GNWs/Ti_3_C_2_T_*x*_ architecture, the GNWs framework improves electrolyte penetration and exposes more Ti_3_C_2_T_*x*_ interfacial/porous regions, allowing deeper utilization of redox-active surface terminations. As a result, ion diffusion and proton-coupled pseudocapacitive processes contribute more significantly to the measured current response, leading to lower apparent *b*-values. Thus, the b-values reflect mixed EDLC/pseudocapacitive kinetics rather than poor charge-storage capability. Furthermore, the following equation can be utilized to ascertain the contribution rates of both capacitance and diffusion processes.

7 $$i\left(v\right)=k_{1}v+k_{2}v^{0.5}$$

As demonstrated in eqs 7, *k*_1_ and *k*_2_ denote constants linked to capacitive and diffusion-controlled mechanisms.^58^ Calculating these values allows for identifying the contributions of each process at a given potential. Figure 6e presents the voltage-dependent diffusion-controlled current (highlighted in red) overlaid on the CV profile of the GNWs/Ti_3_C_2_T_*x*_/Papyex electrode recorded at 20 mV s^–1^. At this scan rate, the contribution of diffusion-controlled capacitance reaches 67.5%. The diffusion contribution for GNWs/Ti_3_C_2_T_*x*_/Papyex was quantified by separating the charge-storage modes across different scan rates (Figure 6f). The dominance of the diffusion-derived fraction at 20 mV s^–1^ means that the charge-storage process is primarily governed by diffusion-limited kinetics. Nevertheless, at a scan rate of 100 mV s^–1^, the diffusion-controlled contribution decreases to 42.5%, indicating a transition toward predominantly capacitive behavior. This attenuation of diffusion-limited processes at elevated scan rates is attributed to kinetic constraints, whereby ions cannot intercalate/deintercalate rapidly enough within the shortened time scale, thereby restricting the diffusion-controlled charge-storage component. It is important to recognize that, although the relative contributions of EDLC and Faradaic pseudocapacitance vary with scan rate, both mechanisms are integral to the overall electrochemical performance of the hybrid composites. The EDLC is promoted by the large accessible surface area and high electronic conductivity of the GNWs and Ti_3_C_2_T_*x*_ frameworks, while the pseudocapacitive contribution is strengthened by the redox-active, surface-terminated functional groups on Ti_3_C_2_T_*x*_. Figure S10a,b summarize the *b*-values obtained for Ti_3_AlC_2_/Papyex and Ti_3_C_2_T_*x*_/Papyex, respectively. In both cases, the *b*-values lie between 0.5 and 1.0, displaying mixed charge-storage kinetics in which the overall capacitance originates from concurrent surface-controlled and diffusion-controlled processes. At a scan rate of 20 mV s^–1^, the diffusion-controlled contributions extracted from the CV profiles (Figure S10c,d) correspond to diffusion fractions of 51.0% for Ti_3_AlC_2_/Papyex and 50.1% for Ti_3_C_2_T_*x*_/Papyex. These values are considerably lower than that of the GNWs/Ti_3_C_2_T_*x*_/Papyex electrode, which manifests a diffusion-controlled contribution of 67.7%. The scan-rate-dependent evolution of the diffusion fraction for Ti_3_AlC_2_/Papyex and Ti_3_C_2_T_*x*_/Papyex is further presented in Figure S10e,f. Comparison across electrodes consistently reflects that, at equivalent scan rates, GNWs/Ti_3_C_2_T_*x*_/Papyex maintains the highest diffusion-controlled contribution. This behavior is attributed to the incorporation of GNWs, which mitigates restacking and effectively enlarges the accessible porosity of the MXene, thereby increasing the electrolyte-electrode contact area. The resulting improvement in ion accessibility enhances utilization of redox-active surface terminations on Ti_3_C_2_T_*x*_, providing a greater population of Faradaic reaction sites and ultimately increasing the pseudocapacitive contribution. Using eq 1, the specific areal capacitance of the various electrodes was determined from the GCD profiles in Figure S9, with the calculated values summarized in Figure 6g. For the GNWs/Ti_3_C_2_T_*x*_/Papyex electrode, areal capacitances of 91.6, 79.5, 69.4, and 53.7 mF cm^–2^ are obtained at current densities of 1.5, 2.0, 2.5, and 3.0 mA cm^–2^, respectively. The gradual decrease in capacitance with increasing current density is attributed to kinetic limitations: At lower current densities, ions have sufficient time to access active sites and complete Faradaic reactions, whereas at higher current densities, the shortened charge–discharge duration restricts ion diffusion and redox utilization, thereby reducing the effective capacitance. The reduced capacitance difference between GNWs/Ti_3_C_2_T_*x*_/Papyex and Ti_3_C_2_T_*x*_/Papyex at higher current density can be explained by this kinetic limitation. At low current density, the GNWs/Ti_3_C_2_T_*x*_/Papyex electrode benefits from improved electrolyte penetration, expanded accessible interfaces, and enhanced utilization of Ti_3_C_2_T_*x*_ surface terminations. When the current density increases, the shortened discharge time restricts ion diffusion into inner porous/interlayer regions and limits the contribution of diffusion-assisted pseudocapacitive sites. Consequently, the additional capacitance provided by the GNWs-enabled hierarchical structure becomes less fully expressed, and the capacitance of GNWs/Ti_3_C_2_T_*x*_/Papyex approaches that of Ti_3_C_2_T_*x*_/Papyex at high current density. Moreover, as demonstrated in Table S1, GNWs/Ti_3_C_2_T_*x*_/Papyex displays superior electrochemical performance in comparison to earlier studies employing MXene electrode materials. To further evaluate the practical charge-storage capability of the electrodes, the areal energy and power densities were calculated from the GCD curves using eqs 3 and 4 at different current densities. As shown in Figure S11a, GNWs/Ti_3_C_2_T_*x*_/Papyex delivers the highest areal energy density among the four tested electrodes across the entire power-density range. The hybrid electrode achieves areal energy densities of approximately 6.23, 5.41, 4.72, and 3.65 μWh cm^–2^ at corresponding areal power densities of 0.52, 0.71, 0.87, and 1.05 mW cm^–2^, respectively. These values are higher than those of Ti_3_C_2_T_*x*_/Papyex, Ti_3_AlC_2_/Papyex, and GNWs/Papyex measured under identical conditions, confirming the improved energy-power balance of the GNWs/Ti_3_C_2_T_*x*_/Papyex electrode. As further shown in the Ragone plot (Figure S11b), the present electrode exhibits excellent output performance, which compares favorably with several previously reported MXene-based electrodes.

To further validate whether a 30 min duration constitutes the optimal growth condition for GNWs, an extended growth period of 50 min was additionally investigated. As shown in Figure S12a, the top-view SEM image of GNWs/Papyex-50 min presents a denser and partially converged surface morphology compared with GNWs/Papyex-30 min, indicating that prolonged ICP-CVD growth does not simply generate more open vertical GNWs. The cross-sectional SEM image depicted in Figure S12b indicates that extending the growth duration from 30 to 50 min does not result in a significant increase in the overall thickness of the GNWs layer. Instead, the GNWs structure can be divided into two regions: an initial vertical growth region close to the Papyex substrate and an upper secondary growth region. The enlarged cross-sectional image in Figure S12c further confirms that the secondary growth mainly occurs near the top part of the GNWs layer, where neighboring GNWs tend to extend laterally, overlap, and form a more compact surface. This morphological evolution partially blocks the open channels formed during the initial vertical growth stage and makes it more difficult for electrolyte ions to penetrate into the inner GNWs network. Therefore, the diminished electrochemical response observed in the 50 min sample is not ascribed to a substantial increase in film thickness; rather, it results from surface convergence and decreased ion accessibility, which are attributable to secondary lateral growth. This interpretation is supported by the CV comparison in Figure S12d. At 30 mV s^–1^ in 1 mol Na_2_SO_4_ solution, GNWs/Papyex-50 min exhibits a clearly smaller enclosed CV area and a lower current density response than GNWs/Papyex-30 min, demonstrating reduced capacitive performance after excessive growth. These results confirm that extending the GNWs growth time beyond 30 min is unfavorable for charge storage, because secondary lateral growth decreases electrolyte accessibility and effective surface utilization. Therefore, a 30 min duration is designated as the optimal growth period for GNWs, offering an appropriate balance among open nanowall morphology, ion-accessible pathways, and capacitive response.

Figure 7a presents the cycling stability of the GNWs/Ti_3_C_2_T_*x*_/Papyex and Ti_3_C_2_T_*x*_/Papyex electrodes as a function of cycle number. The durability was evaluated by continuous GCD cycling for 3000 cycles in 1 M H_2_SO_4_. The GNWs/Ti_3_C_2_T_*x*_/Papyex hybrid exhibits markedly enhanced long-term stability relative to Ti_3_C_2_T_*x*_/Papyex, retaining 81.4% of its initial capacitance after 3000 cycles at 16 mA cm^–2^, thereby demonstrating robust cycling durability. This improved retention can be explained by the incorporation of GNWs, which function as spacers to suppress MXene restacking and lamellar collapse, maintain open ion-transport pathways, and simultaneously provide a mechanically resilient, highly conductive framework that mitigates stress accumulation and limits resistance growth during prolonged cycling. Figures S13a–c present FESEM images of the GNWs/Ti_3_C_2_T_*x*_/Papyex electrode after the cycling stability test at progressively higher magnifications. The micrographs suggest that the MXene-based architecture remains largely preserved, with no discernible volume expansion or severe morphological degradation after prolonged cycling. Consistent with these observations, the EDS spectrum in Figure S13d closely matches that obtained prior to cycling, displaying that the elemental composition is essentially unchanged and that no obvious foreign impurities were introduced into the GNWs/Ti_3_C_2_T_*x*_/Papyex hybrid during the stability test.

**Figure 7:**
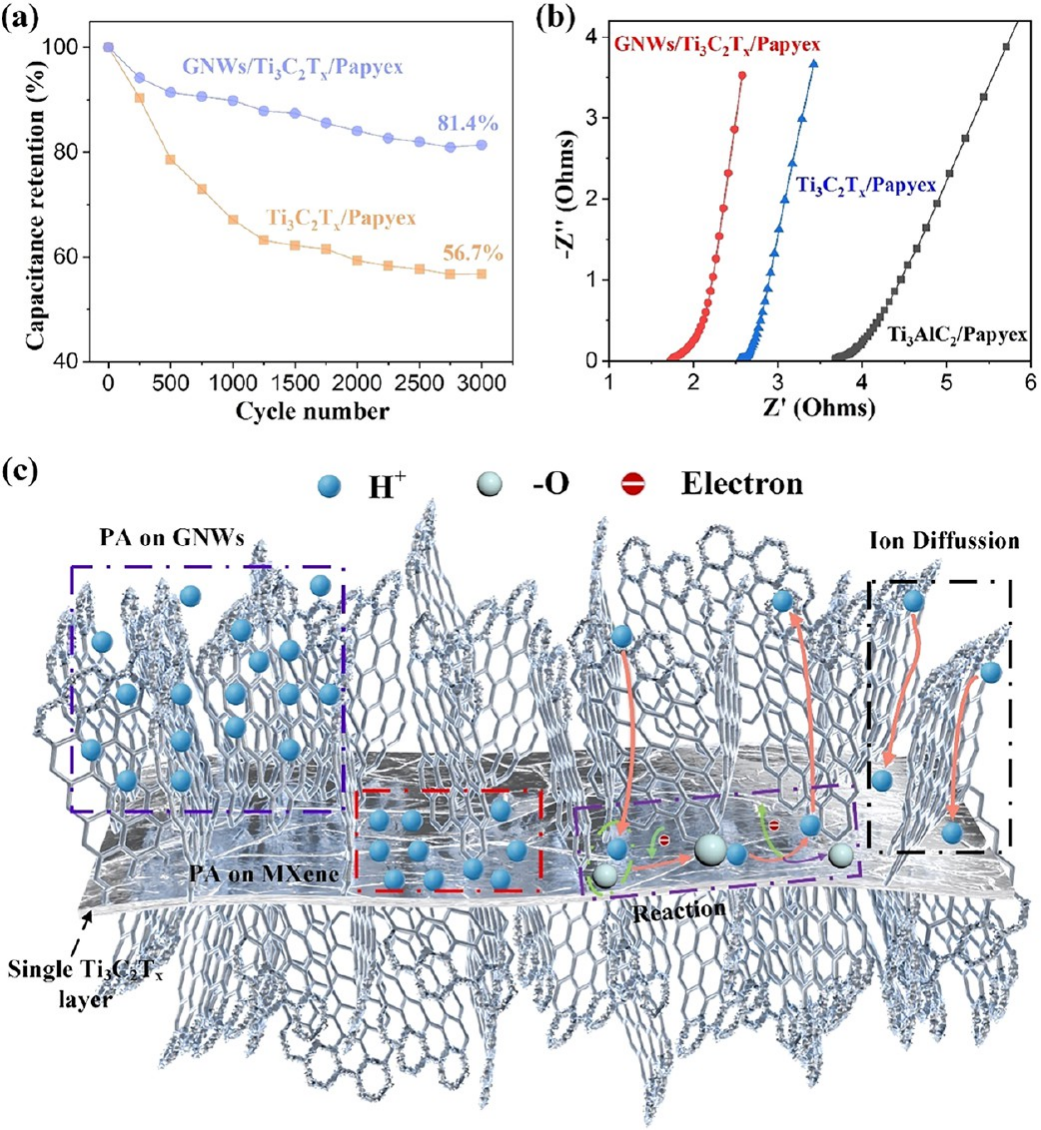
(a) Cyclic ability tests of GNWs/Ti_3_C_2_T_*x*_/Papyex and Ti_3_C_2_T_*x*_/Papyex measured at the current density of 16 mA cm^–2^. (b) Nyquist plots for Ti_3_AlC_2_/Papyex, Ti_3_C_2_T_*x*_/Papyex, and GNWs/Ti_3_C_2_T_*x*_/Papyex, and (c) schematic illustrations of carrier transport strategy in the interfacial region of GNWs/Ti_3_C_2_T_*x*_.

The electrical characteristics of the GNWs/Ti_3_C_2_T_*x*_ hybrid electrodes were evaluated by EIS using a 0.15 V DC bias with a 5 mV AC perturbation over the frequency range of 0.1 Hz to 100 kHz. Figure 7b compares the Nyquist plots of Ti_3_AlC_2_/Papyex, Ti_3_C_2_T_*x*_/Papyex, and GNWs/Ti_3_C_2_T_*x*_/Papyex. To further quantify the resistance characteristics, the EIS spectra were fitted using an equivalent circuit model, as shown in Table S2. In this model, *R*_s_ represents the equivalent series resistance, including electrolyte resistance, intrinsic electrode resistance, and contact resistance, while *R*_ct_ reflects the interfacial charge-transfer resistance. The *R*_s_ fitted values are 3.677, 2.529, and 1.732 Ω for Ti_3_AlC_2_/Papyex, Ti_3_C_2_T_*x*_/Papyex, and GNWs/Ti_3_C_2_T_*x*_/Papyex, respectively. The corresponding *R*_ct_ values are 0.352, 0.195, and 0.126 Ω. The progressive decrease in R_s_ indicates improved series resistance after MXene formation and GNWs incorporation. More importantly, the lowest *R*_ct_ of GNWs/Ti_3_C_2_T_*x*_/Papyex demonstrates faster interfacial charge transfer in the hybrid electrode, supporting the beneficial role of GNWs in improving electrochemical kinetics. On the other hand, in the low-frequency region, the impedance response is dominated by diffusion impedance in the electrolyte, together with interfacial polarization at the working electrode/electrolyte interface, making it particularly sensitive to the electrode architecture.^59^ The inclination of the low-frequency tail therefore provides insight into the charge-transport kinetics of the hybrid electrodes. As shown in Figure 7b, the GNWs/Ti_3_C_2_T_*x*_/Papyex displays the steepest low-frequency slope, exhibiting the most efficient ion/electron transport and the fastest interfacial charge-transfer dynamics among the samples. The GNWs/Ti_3_C_2_T_*x*_/Papyex electrode shows outstanding electrochemical performance, owing to the synergistic effects of its components. Ti_3_C_2_T_*x*_ and GNWs contribute a large specific surface area, while Ti_3_C_2_T_*x*_ features many oxygen functional groups that act as active sites. Each component boosts charge storage capacity without reducing the electrode’s electrical conductivity.

The charge storage in the GNWs/Ti_3_C_2_T_*x*_ hybrid is governed by the combined contribution of EDLC and surface/near-surface pseudocapacitive processes. The enhancement mechanism is schematically represented in Figure 7c. Based on the XPS evidence obtained from directly exposed Ti_3_C_2_T_*x*_/Papyex and the electrochemical response in 1 M H_2_SO_4_, O- and OH-related Ti surface environments are considered to contribute to the pseudocapacitive component through reversible proton-coupled electron transfer (PCET).^60^ In this process, ≡Ti–O sites can be protonated under cathodic polarization to form ≡Ti–OH, while the reverse deprotonation/oxidation occurs during anodic polarization. The corresponding pseudocapacitive process can be represented as

8 $$≡\mathrm{Ti}\text{−}O+H^{+}+e^{-}⇌≡\mathrm{Ti}\text{−}\mathrm{OH}$$

Equation 8 exhibits this reaction that corresponds to the cathodic reduction accompanied by protonation of an O-terminated site (≡Ti–O) to yield a hydroxyl-terminated species (≡Ti–OH), whereas the reverse deprotonation/oxidation proceeds under anodic polarization. For surfaces bearing a mixture of O and OH terminations, the net charge-storage process can be expressed as

9 $${\mathrm{Ti}}_{3}C_{2}O_{x}{\left(\mathrm{OH}\right)}_{y}+{\mathrm{\alpha H}}^{+}+{\mathrm{\alpha e}}^{-}⇌{\mathrm{Ti}}_{3}C_{2}O_{\left(x-\alpha \right)}{\left(\mathrm{OH}\right)}_{\left(y+\alpha \right)}$$

In eq 9, α denotes the fraction of O-type terminations that are converted to OH-type terminations within the applied potential window. Fundamentally, each accessible ≡Ti–O/≡Ti–OH site can participate in a PCET process. Owing to the two-dimensional nature of Ti_3_C_2_T_*x*_, a substantial proportion of Ti atoms are located at or near the surface, resulting in a high areal density of redox-active termination sites. In parallel, both GNWs and Ti_3_C_2_T_*x*_ provide large accessible surface areas that support EDLC via electrostatic physical adsorption (PA) at the electrode/electrolyte interface. In acidic electrolytes, this non-Faradaic contribution is basically linked to the reversible adsorption/desorption of H^+^ on the high-area GNWs and MXene surfaces. Although EDLC does not involve bond formation or electron-transfer reactions, it can contribute substantially to the total capacitance because of the extensive interfacial area provided by the hybrid architecture. Within GNWs/Ti_3_C_2_T_*x*_ hybrid electrodes, GNWs enhance the utilization of both EDLC- and pseudocapacitance-derived sites through three complementary functions. GNWs anchored on MXene sheets act as robust spacers that suppress interlayer collapse and mitigate restacking, thereby maintaining open channels and increasing electrolyte exposure of ≡Ti–O/≡Ti–OH active sites. Their vertically oriented morphology also establishes a 3D conductive framework that accelerates electron transport to termination sites, reduces polarization, and supports rapid PCET kinetics. Meanwhile, the interconnected porous network introduced by GNWs provides continuous ion-transport pathways, shortens proton diffusion distances across the electrode thickness, and promotes electrolyte penetration into interlayer regions, enabling more uniform and deeper participation of active sites. The high density of oxygenated terminations (pseudocapacitance), the large interfacial area (EDLC), and the GNWs-enabled suppression of restacking and transport advantages synergistically improve the capacitance of GNWs/Ti_3_C_2_T_*x*_ electrodes in H_2_SO_4_ electrolyte.

## 4 Conclusions

This work demonstrates the successful fabrication of a GNWs/Ti_3_C_2_T_*x*_/Papyex hybrid electrode with improved supercapacitive performance, enabled by a hierarchical architecture in which GNWs are anchored to Ti_3_C_2_T_*x*_. The hybrid was produced through a sequential route involving HF etching of Ti_3_AlC_2_ to generate Ti_3_C_2_T_*x*_, film assembly on a Papyex substrate via filtration, and subsequent GNWs growth by ICP-CVD. Incorporation of GNWs effectively suppresses MXene restacking, stabilizes multidirectional ion-transport pathways, and increases the fraction of ion-accessible electroactive sites. Simultaneously, the nanostructured GNWs/Ti_3_C_2_T_*x*_ framework enlarges the reactive surface area, thereby improving charge storage relative to the control electrodes. At a scan rate of 10 mV s^–1^, GNWs/Ti_3_C_2_T_*x*_/Papyex delivers an areal capacitance that is 1.2-fold higher than Ti_3_C_2_T_*x*_/Papyex and 7.3-fold higher than GNWs/Papyex. The improvement comes from the synergistic combination: (i) EDLC provided by the high-surface-area GNWs and Ti_3_C_2_T_*x*_ frameworks and pseudocapacitance associated with redox-active surface terminations of Ti_3_C_2_T_*x*_. (ii) The intrinsically high electrical conductivity of GNWs, which promotes rapid electron transport. (iii) Abundant nanostructured interfaces that supply a high density of accessible reaction sites. (iv) GNWs intercalation, which prevents the MXene restacking and self-aggregation, resulting in enhancing exposure and utilization of termination-derived active sites. Essentially, GNWs/Ti_3_C_2_T_*x*_/Papyex composites offer a highly promising development pathway for advancing supercapacitor electrode technology by combining structural stability with optimized ion/electron transport capabilities. This strategy highlights the value of integrating mature technologies with rational nanostructure engineering in energy storage applications.

## Supplementary Material



## Data Availability

Data will be made available on request.

## Notes

ICP-CVD setup; GNWs growth optimization; Raman, XPS, and wettability characterization; electrochemical CV/GCD and charge-storage kinetic analyses; areal energy/power-density and Ragone plots; GNWs overgrowth tests; postcycling FESEM/EDS characterization; comparative electrochemical performance and EIS fitting parameters (PDF)
