# Supplementary material for: Vertical Graphene Nanowalls Anchored on Ti3C2T x MXene as a Hierarchical Composite with Enhanced Supercapacitive Performance
Source: ACS Appl Mater Interfaces. 2026 Jun 26;18(26):36960–76. doi: 10.1021/acsami.6c04344 (PMC13352505; doi:10.1021/acsami.6c04344)
Supplement: Supplementary file 1 [file am6c04344_si_001.pdf]

Supporting Information

Vertical Graphene Nanowalls Anchored on $Ti_3C_2T_x$ MXene as a Hierarchical Composite with Enhanced Supercapacitive Performance

Yang Ma [a b], Ghulam Farid [a b], Shubhadeep Majumdar [a b], Enric Bertran-Serra [a b], Jarosław Serafin [a b c], Stefanos Chaitoglou [* a b], Roger Amade-Rovira [* a b]

[a] Department of Applied Physics, University of Barcelona, C/Martí i Franquès, 1, 08028 Barcelona, Catalunya, Spain

[b] ENPHOCAMAT Group, Institute of Nanoscience and Nanotechnology (IN2UB), University of Barcelona, C/Martí i Franquès, 1, 08028 Barcelona, Catalunya, Spain

[c] Department of Inorganic and Organic Chemistry, Inorganic Chemistry section, University of Barcelona, Martí i Franquès 1-11, 08028, Barcelona, Spain

[*] Corresponding authors

E-mail address: r.amade@ub.edu; stefanoschaitoglou@ub.edu.

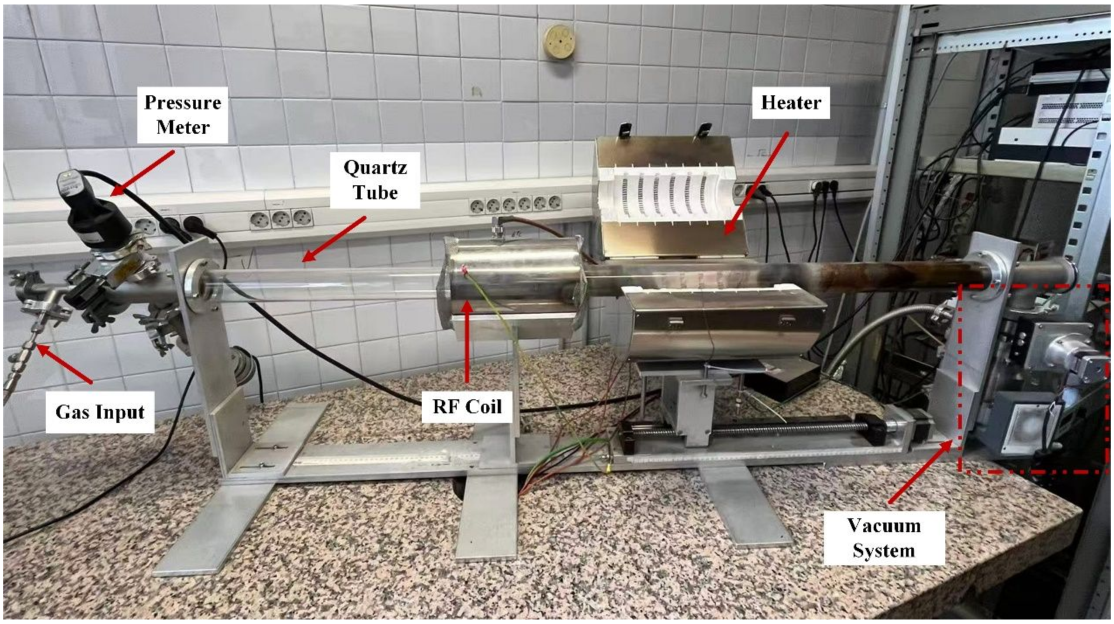

Figure S1. ICP-CVD equipment

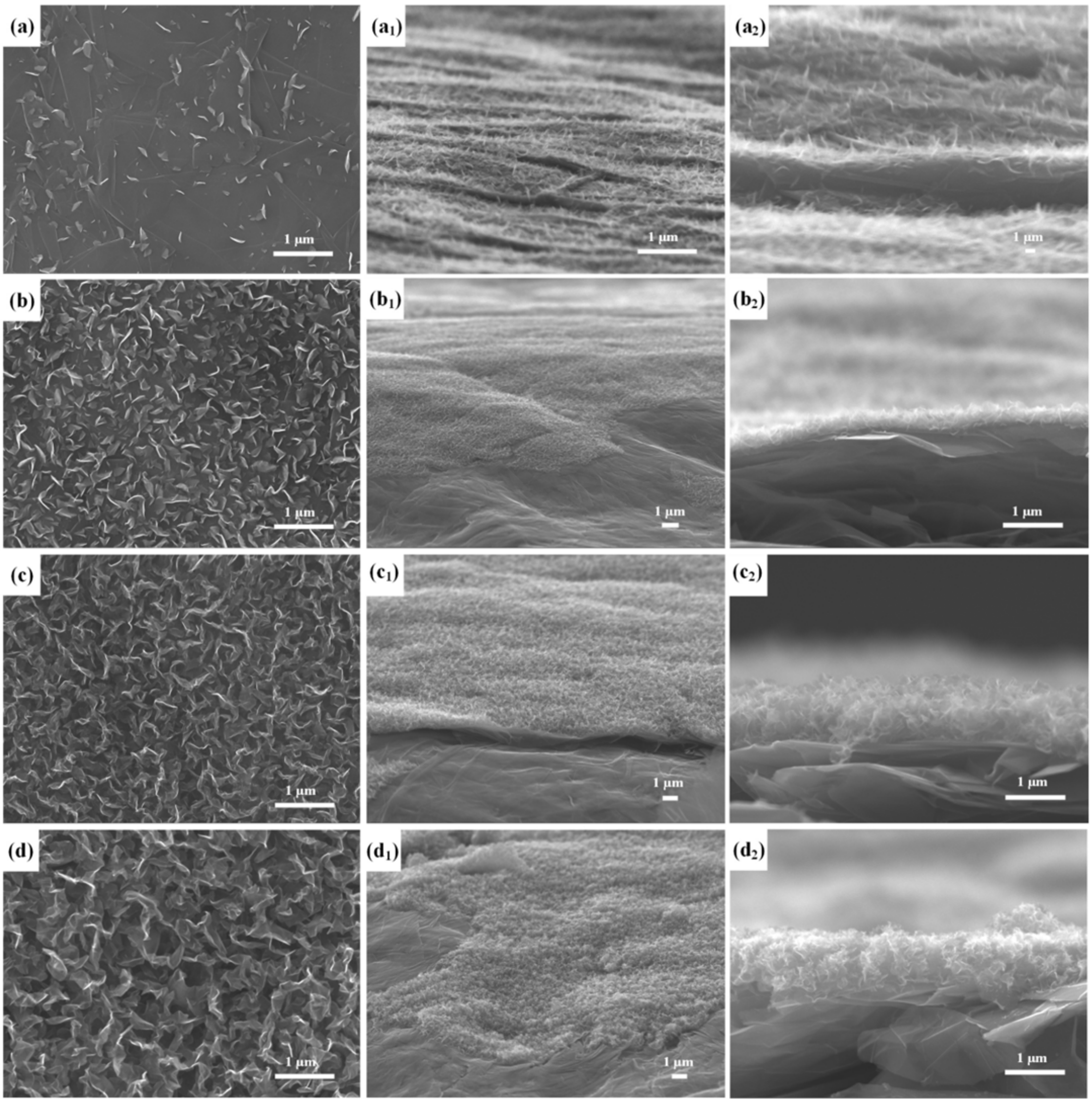

Figure S2. SEM images showing the time-dependent evolution of GNWs grown for 5, 15, 20, and 30 min: (a-d) top-view, (a_1-d_1) 30° tilted-view, and (a_2-d_2) cross-sectional (90°) images.

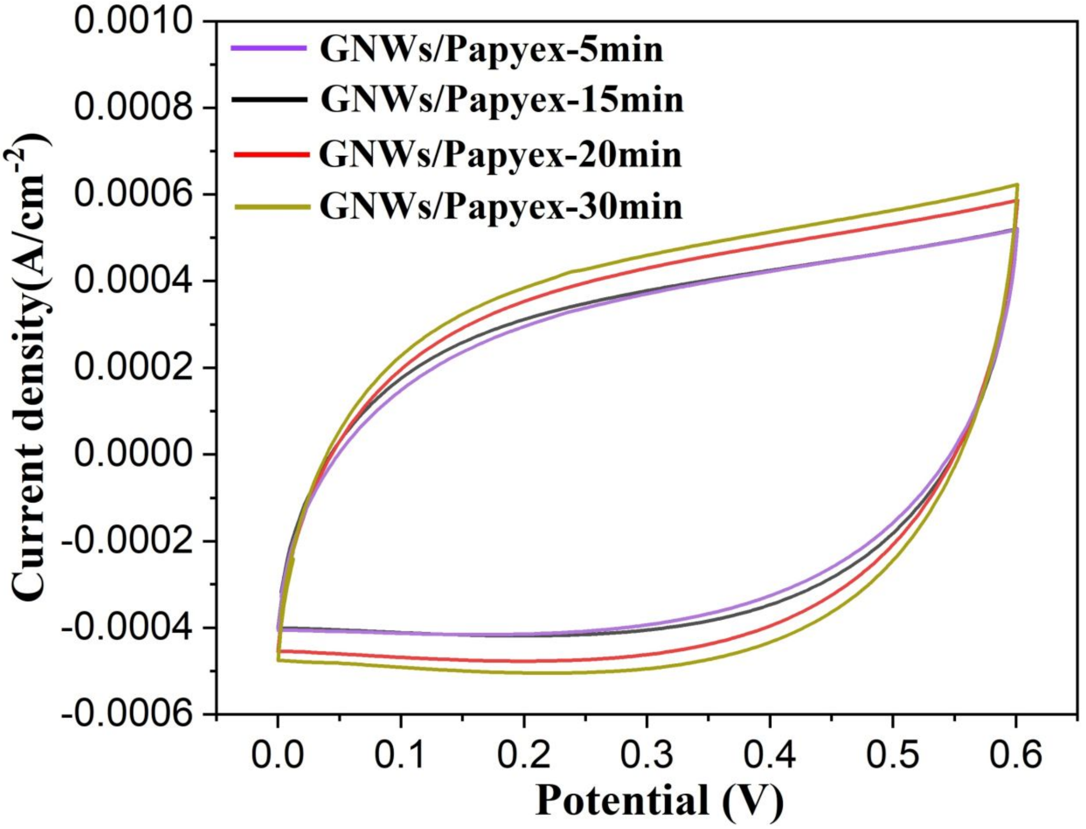

Figure S3. CV curves of GNWs/Papyex electrodes with different growth times of 5, 15, 20, and 30 min, recorded at 10 mV s^{-1}.

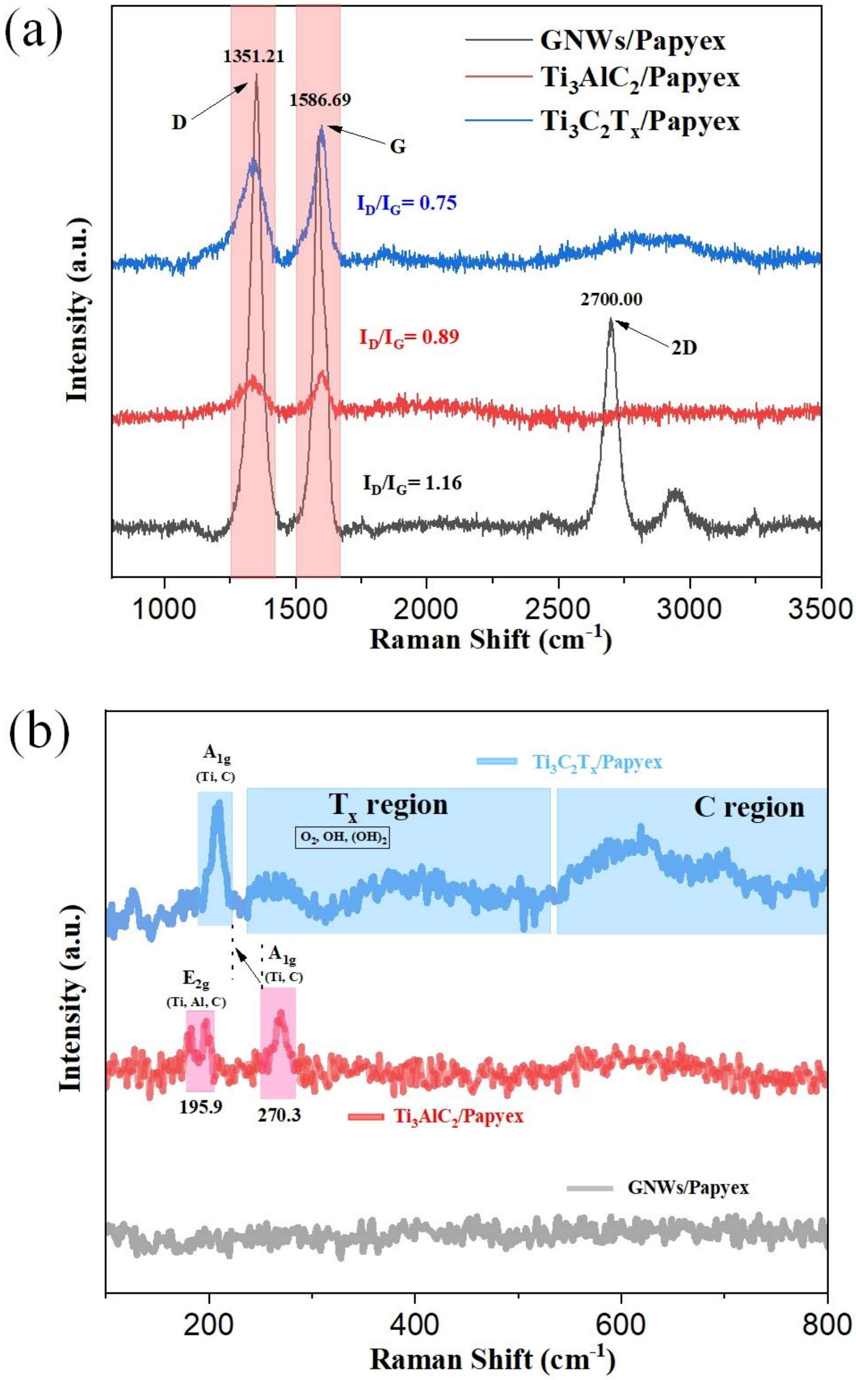

Figure S4. Raman spectra of GNWs/Papyex, Ti_3AlC_2/Papyex, and $Ti_3C_2T_x$/Papyex in the ranges (a) 800-3500 cm^{-1}, and (b) 100-800 cm^{-1}.

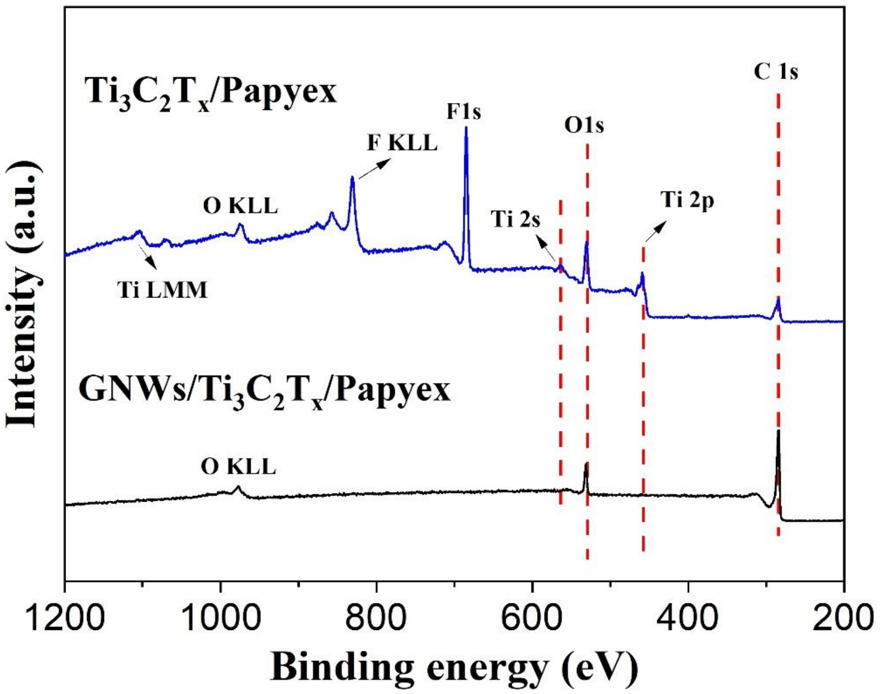

Figure S5. Survey spectra of $Ti_3C_2T_x$/Papyex and GNWs/ $Ti_3C_2T_x$/Papyex.

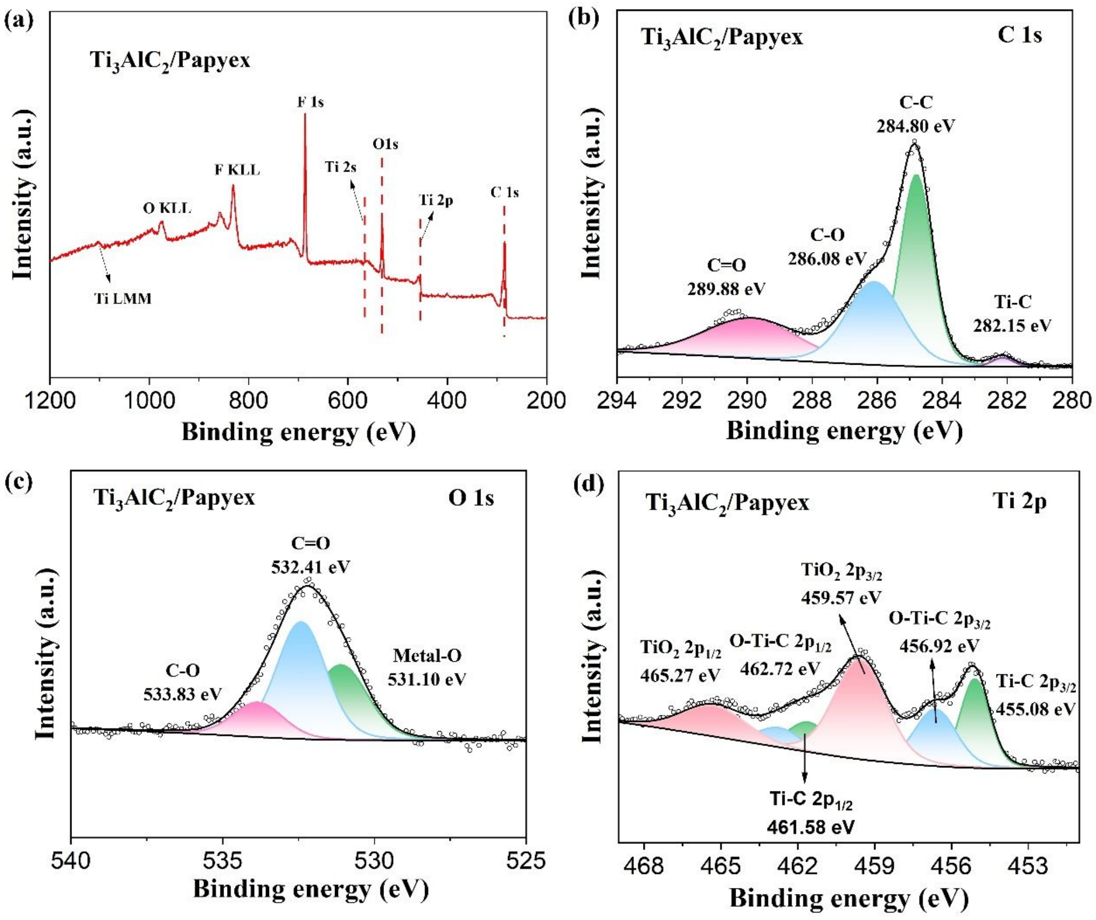

Figure S6. Survey (a) and High-resolution XPS spectrum of (b) C 1s, (c) O 1s, and (d) Ti 2p of the Ti_3AlC_2/Papyex.

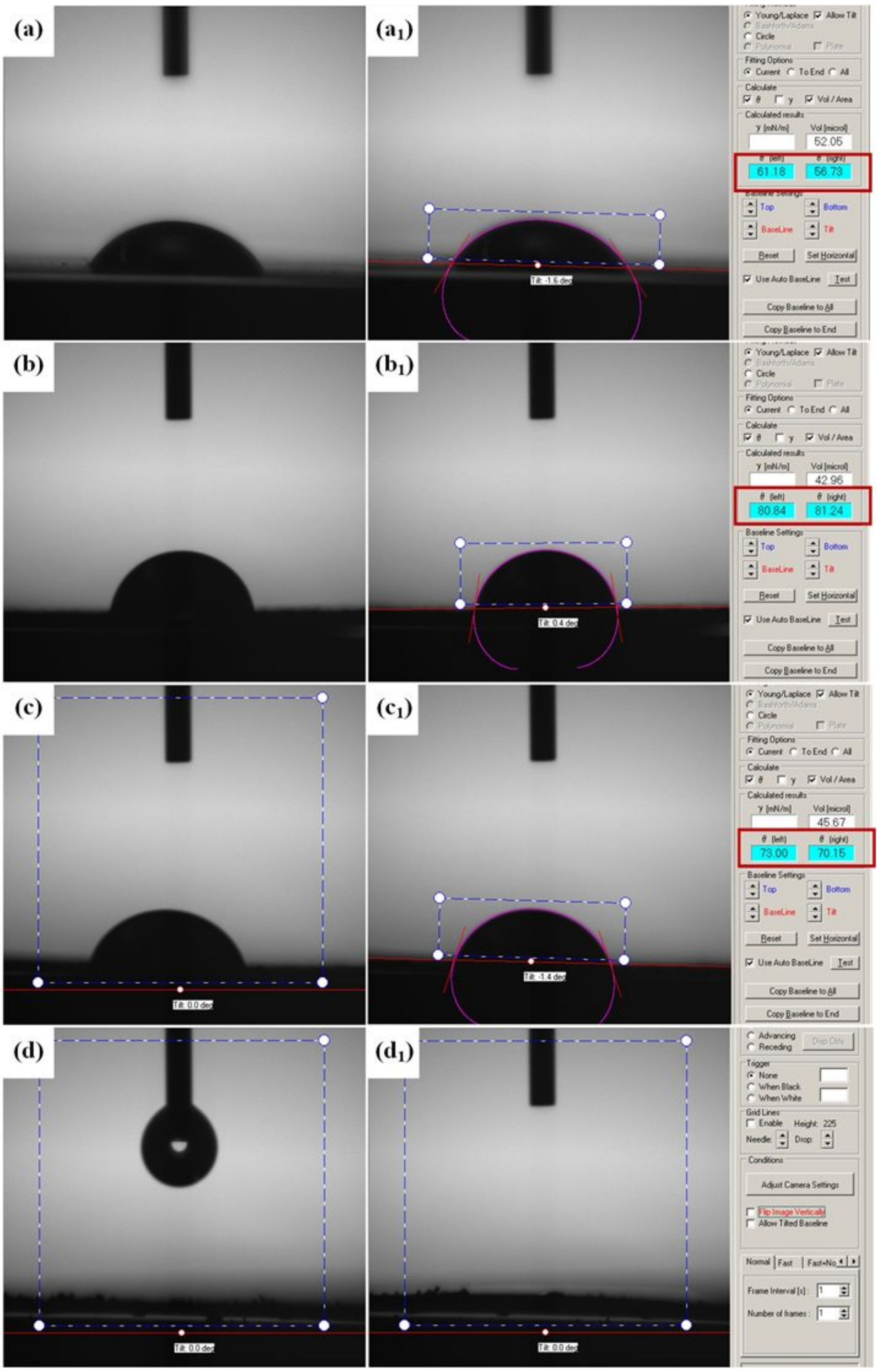

Figure S7. Contact-angle images of (a) Papyex, (b) Ti_3AlC_2/Papyex, (c) $Ti_3C_2T_x$/Papyex, and (d) GNWs/$Ti_3C_2T_x$/Papyex. Reported angles are taken from the left/right fits shown in (a_1-c_1)

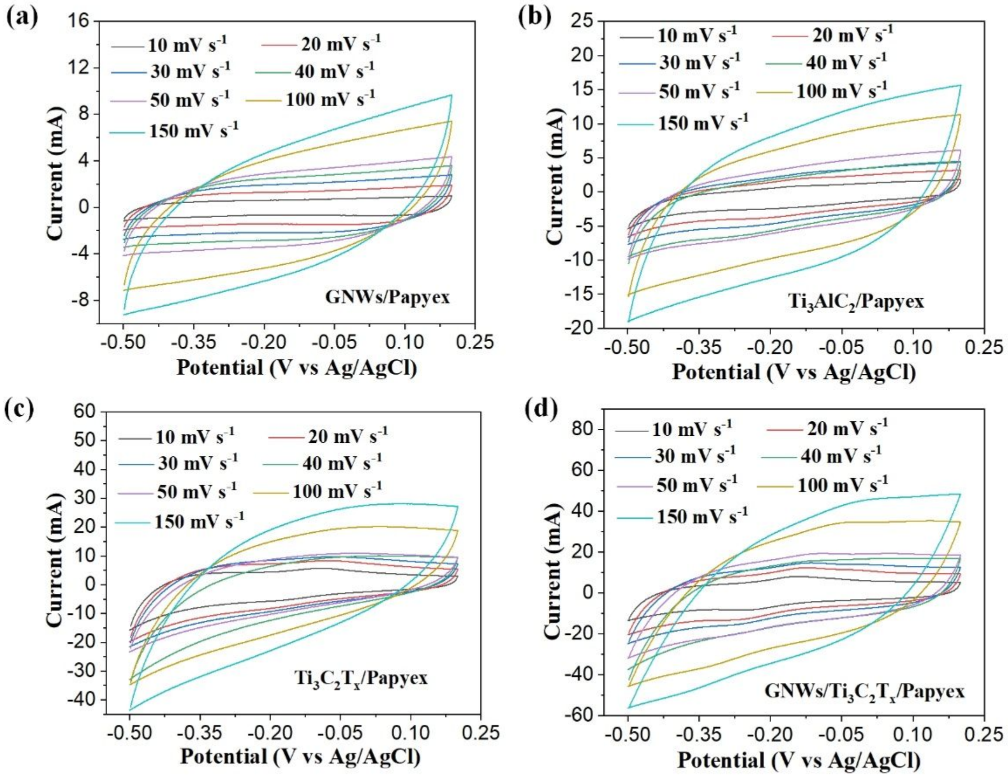

Figure S8. CV measurements of (a) GNWs/Papyex, (b) Ti_3AlC_2/Papyex, (c) $Ti_3C_2T_x$/Papyex, and (d) GNWs/$Ti_3C_2T_x$/Papyex at scan rates from 10 to 150 mV s^{-1}.

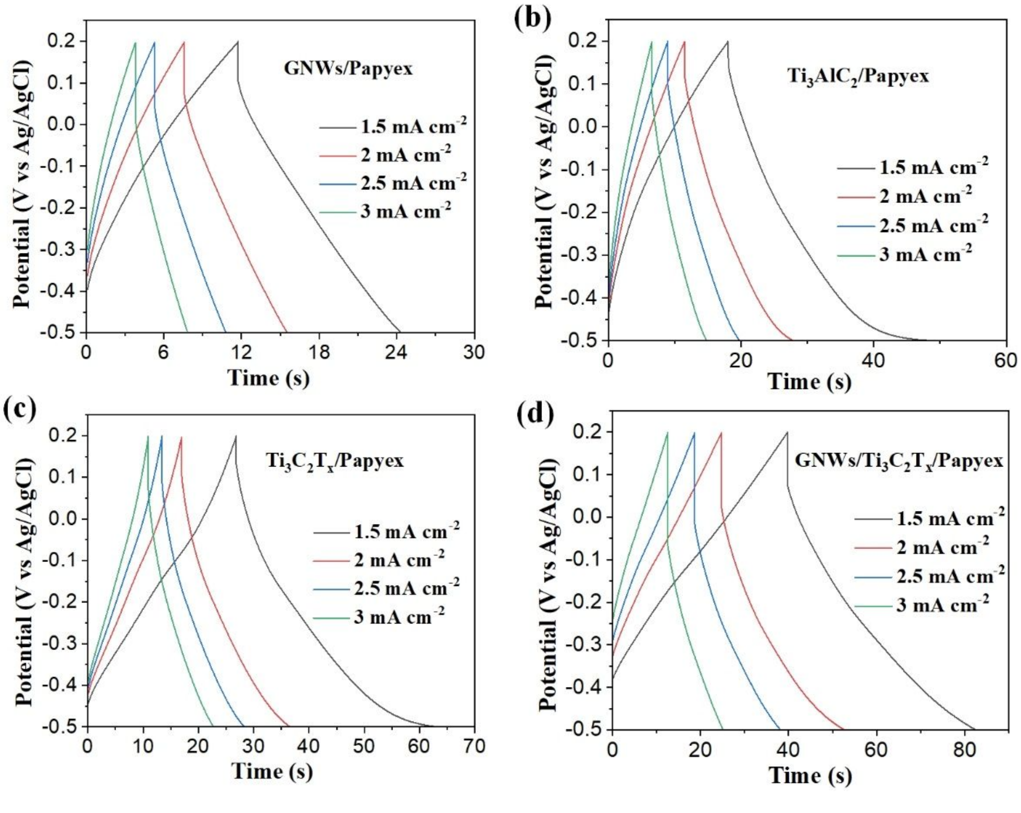

Figure S9. GCD measurements of (a) GNWs/Papyex, (b) Ti_3AlC_2/Papyex, (c)

$Ti_3C_2T_x$/Papyex, and (d) GNWs/$Ti_3C_2T_x$/Papyex at various current densities from 1.5 to 3 mA cm^{-2}.

.

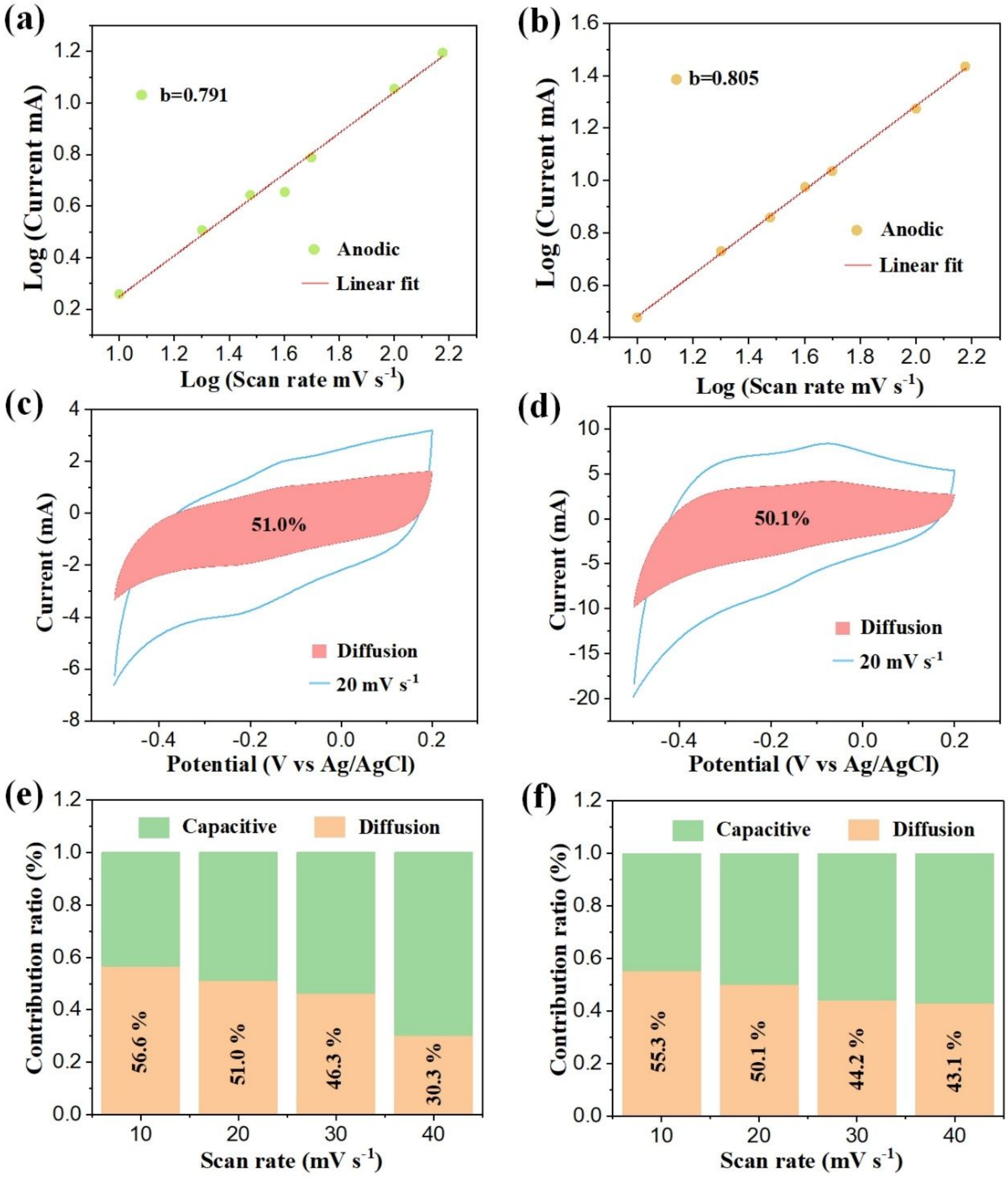

Figure S10. (a) log(i) versus log(v) plots of the current peaks at different scan rates, (c) diffusion contribution curve at 20 mV s^{-1}, (e) the normalized diffusion contribution proportion at each scan rate for the Ti_3AlC_2/Papyex electrode. (b) log(i) versus log(v) plots of the current peaks at different scan rates, (d) diffusion contribution curve under 20 mV s^{-1}, (f) the normalized diffusion contribution proportion at each scan rate for the $Ti_3C_2T_x$/Papyex electrode.

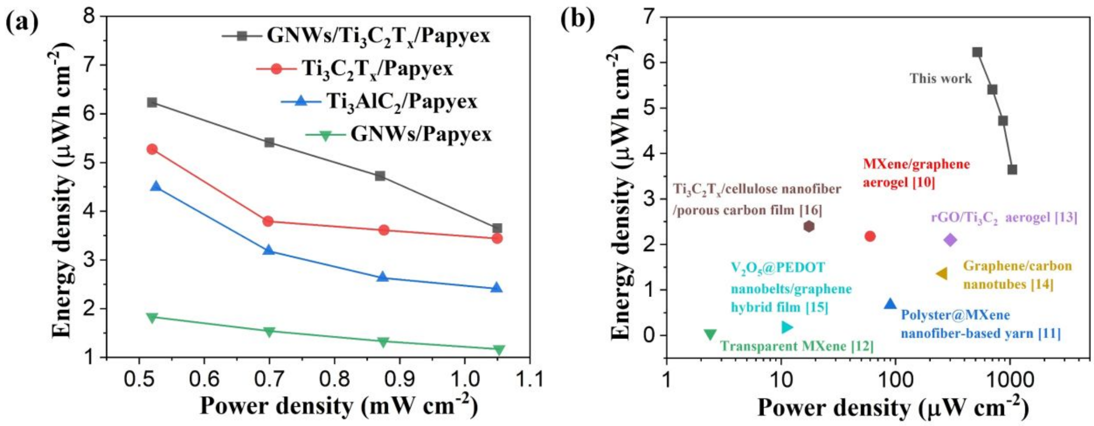

Figure S11. (a) Areal energy density and areal power density of GNWs/Papyex, Ti_3AlC_2/Papyex, $Ti_3C_2T_x$/Papyex, and GNWs/$Ti_3C_2T_x$/Papyex calculated from GCD curves at different current densities. (b) Ragone plot comparing GNWs/$Ti_3C_2T_x$/Papyex with representative MXene-based electrodes reported in the literature.

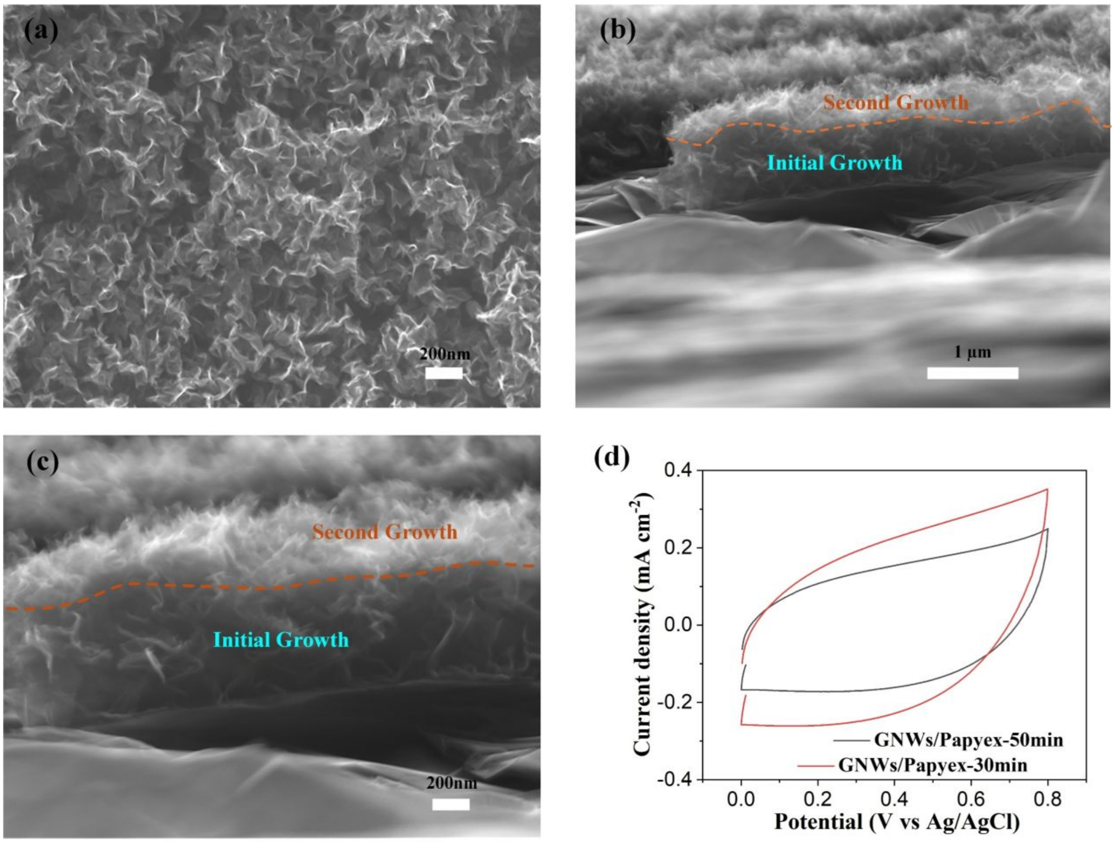

Figure S12. Morphological and electrochemical comparison of GNWs/Papyex grown for extended duration: (a) top-view SEM image of GNWs/Papyex-50min; Cross-sectional SEM images showing the initial vertical growth region and secondary overgrowth region with (b) low magnification and (c) high magnification; (d) CV curves of GNWs/Papyex-30 min and GNWs/Papyex-50 min measured at 30 mV s^{-1} in Na_2SO_4 solution.

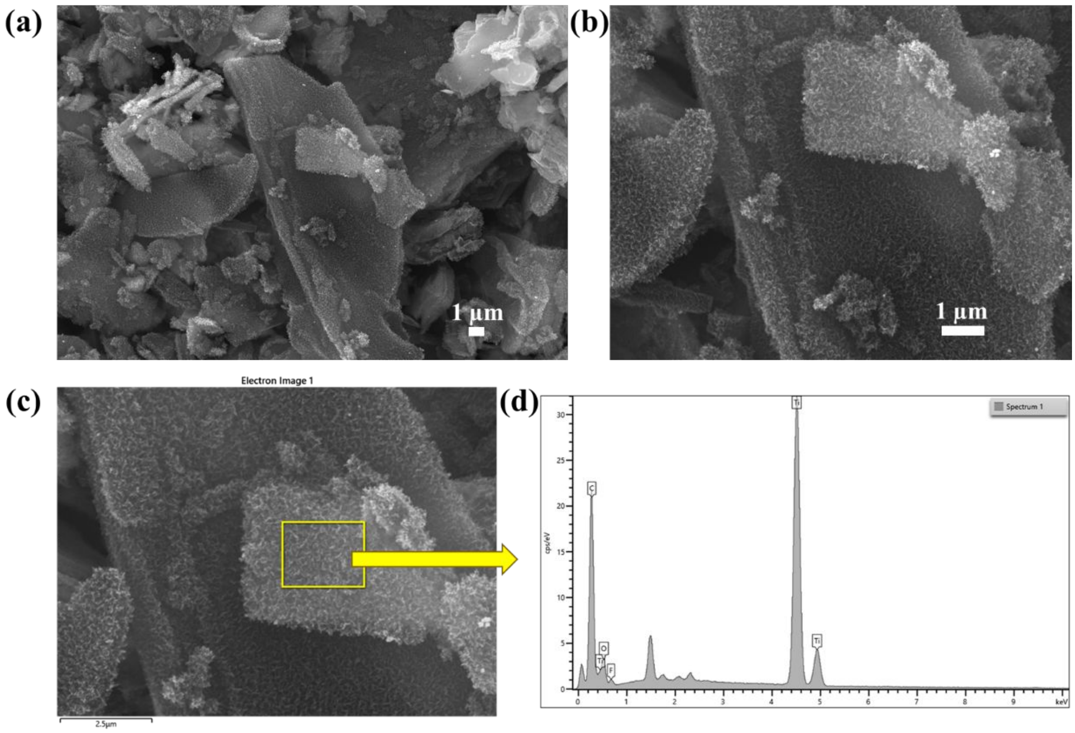

Figure S13. (a), (b), and (c) The FESEM images of GNWs/$Ti_3C_2T_x$/Papyex electrode with different test zones; and (d) EDS spectrum of the GNWs/$Ti_3C_2T_x$/Papyex after cyclic stability test.

Table S1. Electrochemical performance of the GNWs/$Ti_3C_2T_x$/Papyex with reported materials.

Materials	Electrolyte	Potential range (V)	Areal capacitance (mF cm^{-2})	Refs.
GNWs/$Ti_3C_2T_x$/Papyex	1M H_2SO_4	-0.5~0.2	163.2 at 10 mV s^{-1}	This work
MXene-$BiFeO_3$-ZnO	Sodium Sulfate + NaCMC polymer	0~0.5	142.8 at 7 μA cm^{-2}	1
PP-supported Mxene	6M KOH	-1~0	82.6 at 5 mV s^{-1}	2
MXene textile	LiCl/PVA	0~6	146.0 at 0.16 mA cm^{-2}	3
Be^{2+}-MXene	1.0 M Li_2SO_4	-1.2~-0.4	77.2 at 10 mV s^{-1}	4
Mn^{2+}-MXene	PVA/H_3PO_4	0~1	87.0 at 2 mV s^{-1}	5
MXene-TiO_{2}.$CuTiO_3$	2M KOH	0~0.6	144.3 at 15 mA cm^{-2}	6
V_2CT_x	2 M $ZnSO_4$	0.1~1.6	54.1 at 0.1 mA cm^{-2}	7
PVA/SA/MXene	hydrogel $S_{0.2}/M_3/P\text{-}N_{0.3}$	0~1	130.8 at 1 mA cm^{-2}	8
$Ti_3C_2PBr_x$ MXene	1M H_2SO_4	-0.35~0.2	360.0 at 20 mV s^{-1}	9

Table S2. Fitted resistance parameters obtained from the EIS spectra.

**Sample**	**Equivalent circuit model**	**R_s (Ω)**	**R_{ct} (Ω)**
Ti_3AlC_2/Papyex	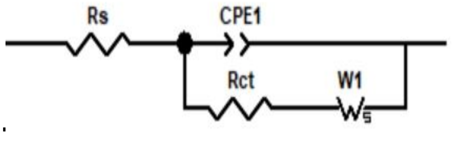	3.677	0.352
$Ti_3C_2T_x$/Papyex		2.529	0.195
GNWs/$Ti_3C_2T_x$/Papyex		1.732	0.126

(1) Nag, R.; Das, S.; Das, D.; Venimadhav, A.; Bera, A. A MXene-$BiFeO_3$-ZnO Nanocomposite Photocatalyst Served as a High-Performance Supercapacitor Electrode. *Phys. Chem. Chem. Phys.* **2023**, 25 (34), 23125-23132.
(2) Garg, R.; Agarwal, A.; Agarwal, M. Synthesis and Optimisation of MXene for Supercapacitor Application. *J. Mater. Sci.: Mater. Electron.* **2020**, *31* (21), 18614-18626.
(3) Inman, A.; Hryhorchuk, T.; Bi, L.; Wang, R.; Greenspan, B.; Tabb, T.; Gallo, E. M.; VahidMohammadi, A.; Dion, G.; Danielescu, A.; Gogotsi, Y. Wearable Energy Storage with MXene Textile Supercapacitors for Real World Use. *J. Mater. Chem. A* **2023**, *11* (7), 3514-3523.
(4) Li, S.; Shi, Q.; Li, Y.; Yang, J.; Chang, T.-H.; Jiang, J.; Chen, P.-Y. Intercalation of Metal Ions into $Ti_3C_2T_x$ MXene Electrodes for High-Areal-Capacitance Microsupercapacitors with Neutral Multivalent Electrolytes. *Adv. Funct. Mater.* **2020**, *30* (40), 2003721.
(5) Feng, X.; Ning, J.; Wang, B.; Guo, H.; Xia, M.; Wang, D.; Zhang, J.; Wu, Z.-S.; Hao, Y. Functional Integrated Electromagnetic Interference Shielding in Flexible Micro-Supercapacitors by Cation-Intercalation Typed $Ti_3C_2T_x$ MXene. *Nano Energy* **2020**, *72*, 104741.
(6) Noman, M.; Mahmood Baig, M.; Muhammad Saqib, Q.; Patil, S. R.; Patil, C. S.; Kim, J.; Ko, Y.; Lee, E.; Hwang, J.; Goo Lee, S.; Bae, J. $Ti_3C_2T_x$-MXene based 2D/3D Ti_3C_2-TiO_2-$CuTiO_3$ Heterostructure for Enhanced Pseudocapacitive Performance. *Chem. Eng. J.* **2024**, *499*, 156697.
(7) Zhao, S.; Luo, X.; Cheng, Y.; Shi, Z.; Huang, T.; Yang, S.; Zheng, H.; Bi, Y.; Zhang, J.; Shi, Q.; Cao, M.; Zhang, C.; Yue, Y.; Ma, Y. A flexible Zinc Ion Hybrid Capacitor Integrated System with Layers-Dependent V_2CT_x MXene. *Chem. Eng. J.* **2023**, *454*, 140360.
(8) Tian, J.; Sun, Z.; Shi, C.; Huang, Z. Rapid Fabrication of Tough Sodium Alginate/MXene/Poly(Vinyl Alcohol) Dual-Network Hydrogel Electrolytes for Flexible All-Solid-State Supercapacitors. *Int. J. Biol. Macromol.* **2023**, *248*, 125937.
(9) Zhu, J.; Zhu, S.; Cui, Z.; Li, Z.; Wu, S.; Xu, W.; Gao, Z.; Ba, T.; Liang, C.; Liang, Y.; Jiang, H. Dual Redox Reaction Sites for Pseudocapacitance Based on Ti and -P

Functional Groups of $Ti_3C_2PBr_x$ MXene. *Angew. Chem.* **2024**, *136* (27), e202403508.
(10) Yue, Y.; Liu, N.; Ma, Y.; Wang, S.; Liu, W.; Luo, C.; Zhang, H.; Cheng, F.; Rao, J.; Hu, X.; Su, J.; Gao, Y. Highly Self-Healable 3D Microsupercapacitor with MXene-Graphene Composite Aerogel. *ACS Nano* **2018**, *12* (5), 4224-4232.
(11) Shao, W.; Tebyetekerwa, M.; Marriam, I.; Li, W.; Wu, Y.; Peng, S.; Ramakrishna, S.; Yang, S.; Zhu, M. Polyester@MXene Nanofibers-Based Yarn Electrodes. *J. Power Sources* **2018**, *396*, 683-690.
(12) Zhang, C.; Anasori, B.; Seral-Ascaso, A.; Park, S.-H.; McEvoy, N.; Shmeliov, A.; Duesberg, G. S.; Coleman, J. N.; Gogotsi, Y.; Nicolosi, V. Transparent, Flexible, and Conductive 2D Titanium Carbide (MXene) Films with High Volumetric Capacitance. *Adv. Mater.* **2017**, *29* (36), 1702678.
(13) N, R.; A, K.; H.M, M.; M.R, N.; Mondal, D.; Nataraj, S. K.; Ghosh, D. Binder Free Self-Standing High Performance Supercapacitive Electrode Based on Graphene/Titanium Carbide Composite Aerogel. *Appl. Surf. Sci.* **2019**, *481*, 892-899.
(14) Wang, Y.; Zhang, Y.; Wang, G.; Shi, X.; Qiao, Y.; Liu, J.; Liu, H.; Ganesh, A.; Li, L. Direct Graphene-Carbon Nanotube Composite Ink Writing All-Solid-State Flexible Microsupercapacitors with High Areal Energy Density. *Adv. Funct. Mater.* **2020**, *30* (16), 1907284.
(15) Wang, L.; Shu, T.; Guo, S.; Lu, Y.; Li, M.; Nzabahimana, J.; Hu, X. Fabricating Strongly Coupled V_2O_5@PEDOT Nanobelts/Graphene Hybrid Films with High Areal Capacitance and Facile Transferability for Transparent Solid-State Supercapacitors. *Energy Storage Mater.* **2020**, *27*, 150-158.
(16) Chen, W.; Zhang, D.; Yang, K.; Luo, M.; Yang, P.; Zhou, X. Mxene ($Ti_3C_2T_x$)/Cellulose Nanofiber/Porous Carbon Film as Free-Standing Electrode for Ultrathin and Flexible Supercapacitors. *Chem. Eng. J.* **2021**, *413*, 127524.
